# Tumor-specific MHC-II guides anthracycline exemption and immunotherapy benefit in breast cancer

**DOI:** 10.1186/s40364-025-00797-9

**Published:** 2025-06-10

**Authors:** Zehao Wang, Yanhui Wang, Zhishuang Gao, Yue Zhou, Xiaoting Chen, Rui Xu, Yangsiyuan Zhao, Yi Zhang, Bingqiu Xiu, Jing Liu, Zhiming Shao, Shengmei Gu, Jingyan Xue, Jiong Wu

**Affiliations:** 1 Department of Breast Surgery, Key Laboratory of Breast Cancer, Fudan University Shanghai Cancer Center, Shanghai, 200032 China; 2 Department of Oncology, Shanghai Medical College, Fudan University, Shanghai, 200032 China

**Keywords:** Triple-negative breast cancer, MHC-II, Anthracycline exemption, Immunotherapy, KAT2B

## Abstract

**Background:**

Anthracycline-based chemotherapy, while foundational in breast cancer treatment, confers substantial cardiotoxicity. Identifying biomarkers to guide anthracycline exemption without compromising efficacy has remained an unresolved clinical challenge for decades.

**Methods:**

We conducted multi-cohort spatial-omics and clinical validation integrating 345 early-stage triple-negative breast cancer (eTNBC) and 167 HER2 + breast cancer patients from Fudan University Shanghai Cancer Center (FUSCC) cohorts, alongside 150 eTNBC patients from a validation cohort. Tumor-specific MHC-II (tsMHC-II) expression was quantified via multiplex immunohistochemistry (mIHC). Mechanistic insights were derived from the NeoTRIP immunotherapy spatial cohort, I-SPY2 trial data, TCGA database, ATAC-seq chromatin profiling, ChIP, and patient-derived organoid (PDO)-immune cell co-culture systems.

**Results:**

In eTNBC, high tsMHC-II expression predicted improved disease-free survival (DFS) and comparable overall survival (OS) with paclitaxel-carboplatin (PCb) versus anthracycline-sequential paclitaxel (EC-P), identifying tsMHC-II as a predictive marker for anthracycline exemption. High tsMHC-II correlated with prolonged DFS and OS in both TNBC and HER2 + subtypes. Multi-omics including spatial and transcriptional cohorts revealed tsMHC-II-high tumors harbor immune-rich microenvironments with elevated cytotoxic T cells, B cells, and antigen-presenting cells. Validation in NeoTRIP and I-SPY2 cohorts demonstrated superior immunotherapy response in tsMHC-II-high patients. Mechanistically, ATAC-seq, ChIP and PDO co-culture models confirmed that KAT2B upregulated tsMHC-II via CIITA promoter acetylation, sustaining immunotherapeutic vulnerability.

**Conclusion:**

TsMHC-II serves as a dual biomarker for adjuvant anthracycline chemotherapy exemption and neoadjuvant immunotherapy stratification in TNBC, driven by KAT2B-mediated epigenetic remodeling. These findings advance precision strategies to reduce anthracycline toxicity while enhancing immune activation in eTNBC.

**Supplementary Information:**

The online version contains supplementary material available at 10.1186/s40364-025-00797-9.

## Introduction

For over half a century, anthracycline-based regimens have been central to adjuvant chemotherapy in breast cancer, significantly improving survival outcomes [1–4]. However, their cumulative cardiotoxicity and risk of secondary malignancies have intensified debates about whether subsets of patients can safely forgo anthracyclines without compromising efficacy [5–8]. While HER2 + breast cancer trials, such as BCIRG-006, demonstrated comparable outcomes between anthracycline-free (PCb) and anthracycline-containing (EC-P) regimens under anti-HER2 therapy [9], TNBC lacks consensus. The PATTERN study found no difference in OS between PCb regimen and EC-P regimen, suggesting the possibility of anthracycline exemption in postoperative adjuvant chemotherapy for TNBC [10]. TOP2A amplification was once considered a potential marker for anthracycline exemption in early breast cancer; however, the READ study, through a large prospective cohort, confirmed that TOP2A amplification did not effectively identify breast cancer patients who could achieve equivalent overall survival outcomes without anthracycline treatment [11, 12], underscoring the unmet need for reliable predictive biomarkers for anthracycline exemption in postoperative TNBC treatment.

The advent of immunotherapy in early-stage TNBC (eTNBC) further complicates this landscape [12–19]. Trials like NeoPACT and NeoTRIP reported promising pathological complete response (pCR) rates (58% and 48.6%, respectively) and survival outcomes with anthracycline-free chemoimmunotherapy [20, 21]. However, these results lag behind anthracycline-containing regimens, such as KEYNOTE-522 (pCR: 64.8%) [13, 22, 23], emphasizing the necessity of biomarkers to identify patients who can optimally benefit from combined anthracycline exemption and immune activation.

Whether patients with high immunotherapy response potential can be exempted from anthracycline chemotherapy effects poses a critical question regarding how to identify patients who would derive substantial benefit from immunotherapy. Tumor-specific MHC-II (tsMHC-II) emerges as a pivotal candidate. Beyond its prognostic value across cancers [24–28], existing studies in vivo have shown that tsMHC-II, compared to tsMHC-I, can present a broader range of tumor-associated neoantigens, playing a vital part in reshaping anti-tumor immune microenvironment(TME), activating CD4 + T cell anti-tumor effects by using tumor necrosis factor–α and Fas ligand (FasL), and inducing more stringent environmental pressures for tumor survival evolution [29–31]. Moreover, in mouse models, tsMHC-II can mediate spontaneous rejection of tumor-associated neoantigens, confirming that tsMHC-II plays a crucial role in the immune recognition of tumor antigens [32]. Notably, several eTNBC neoadjuvant chemo-immunotherapy (NAC-IT) trials, such as I-SPY2 and the NeoTRIP spatial cohorts, have confirmed that high expression of tsMHC-II predicts better NAC-IT treatment outcomes and is a potential benefit stratification marker for eTNBC. Additionally, the NeoTRIP spatial cohort has confirmed that tumor subgroups with high MHC-II expression have better predictive efficacy for immunotherapy response compared to PD-L1 positive tumors [33, 34]. In a clinical cohort of 30 melanoma patients, high tsMHC-II expression was associated with improved response rates to anti-PD-1/PDL1 immunotherapy and enhanced survival benefits, demonstrating superior predictive efficacy for immunotherapy response compared to PD-L1 [25]. Concurrently, a validation study in bladder cancer based on MHC-II pathway expression confirmed that the MHC-II pathway exhibits higher predictive efficacy for anti-PD-L1 immunotherapy response than tumor mutation burden (TME) [35]. However, existing research lacks both systematic comparisons between tsMHC-II and established immunotherapy benefit predictors such as PD-L1 and TILs in large clinical immunotherapy cohorts, as well as exploration of anthracycline chemotherapy de-escalation from the perspective of immunotherapy predictive biomarkers like tsMHC-II. Paradoxically, although anthracycline induces immunogenic cell death induction, it exerts myelosuppressive effects and damps systemic immunity [36]. This duality highlights the urgency to define biomarkers that reconcile anthracycline sparing with sustained immune activation.

This study interrogated tsMHC-II’s dual role in guiding anthracycline exemption and immunotherapy stratification. Using spatial mIHC across 495 TNBC patients (FUSCC exploratory and validation cohorts), we demonstrated that high tsMHC-II expression confers survival parity between PCb and EC-P, supporting anthracycline exemption. Integrated multi-omics (mIHC, RNA-seq, NeoTRIP cohort, I-SPY2 cohort, TCGA) revealed tsMHC-II’s association with immune-rich microenvironments and superior immunotherapy response. Mechanistically, ATAC-seq, ChIP and PDO co-cultures identified KAT2B as an epigenetic regulator of tsMHC-II via CIITA promoter acetylation, sustaining immunotherapeutic vulnerability. This work positions tsMHC-II as a dual-functional biomarker, enabling “de-escalation and immune activation” strategies to mitigate anthracycline toxicity while optimizing outcomes in eTNBC.

## Results

### The exploration study cohort

**Fig. 1:**
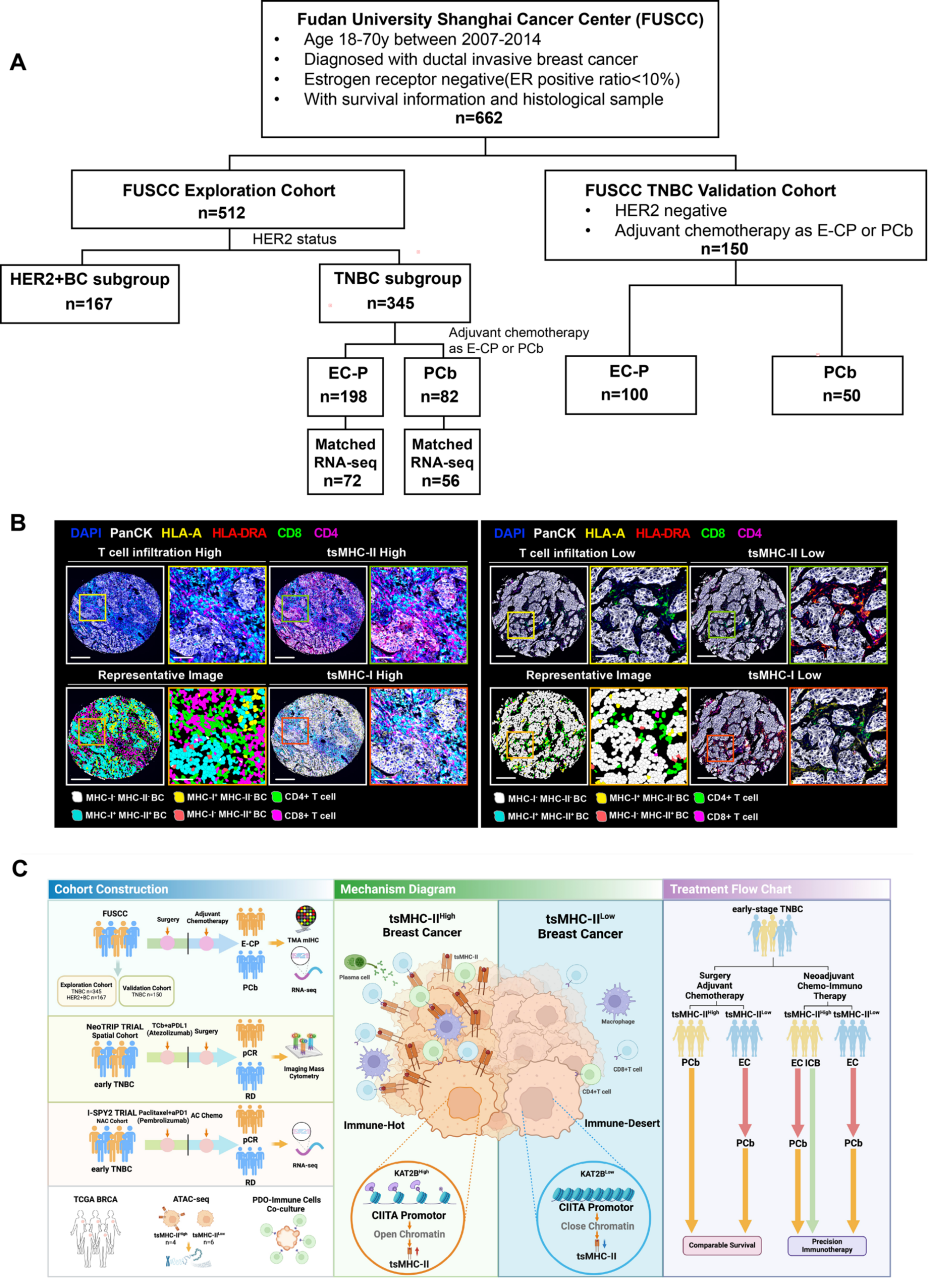
Study Cohort and tsMHC-II-guided Clinical Anthracycline Exemption Treatment Flowchart. **A**. Flowchart of patient inclusion in the FUSCC exploratory and validation cohorts. **B**. Schematic diagram of the FFPE tissue microarray mIHC panel, showing high and low expression groups for tsMHC-II, tsMHC-I, CD4, and CD8. High magnification: Scale bar = 250µm; low magnification: Scale bar = 50µm. **C**. Schematic diagram of the cohort construction, mechanism diagram and clinical  treatment flow chart.

This study enrolled 662 patients from Fudan University Shanghai Cancer Center between 2007 and 2014, including an exploratory cohort of 512 patients with TNBC or HER2-positive breast cancer, and a validation cohort of 150 TNBC patients. The exploratory cohort comprised 345 TNBC cases and 167 HER2-positive cases. Among TNBC patients receiving postoperative adjuvant chemotherapy, 82 were treated with paclitaxel-carboplatin (PCb) and 198 with anthracycline followed by paclitaxel (EC-P). RNA-seq data from primary tumors were available for 56 PCb-treated and 72 EC-P-treated patients. Meanwhile, the TNBC validation cohort included 100 patients receiving EC-P and 50 receiving PCb as adjuvant chemotherapy (Fig. 1A).

All patients underwent standardized surgical treatment and had complete clinical-pathological data and survival follow-up information (with a median follow-up time over 5 years) (Table S1). We evaluated tumor specific MHC-I (tsMHC-I), tsMHC-II positive proportion of all the detected tumor cells in each patient, and the proportion of CD4 + and CD8 + T cell infiltration in each patient using multiplex immunohistochemistry (mIHC) (PanCK, HLA-A, HLA-DR, CD4, CD8, and DAPI) (Fig. 1B). This study adheres to the standards and requirements of the REporting recommendations for tumour MARKer prognostic studies (REMARK) [37]. Summary of cohort construction, mechanism diagram, and treatment flow chart (Fig. 1C).

### High expression of tsMHC-II predicts better DFS and OS in breast cancer

In this study, tsMHC-II high expression was defined as  >5% tsMHC-II-positive cells [33, 38], while tsMHC-I, CD4, and CD8 were categorized into high and low expression groups by median values.

**Fig. 2:**
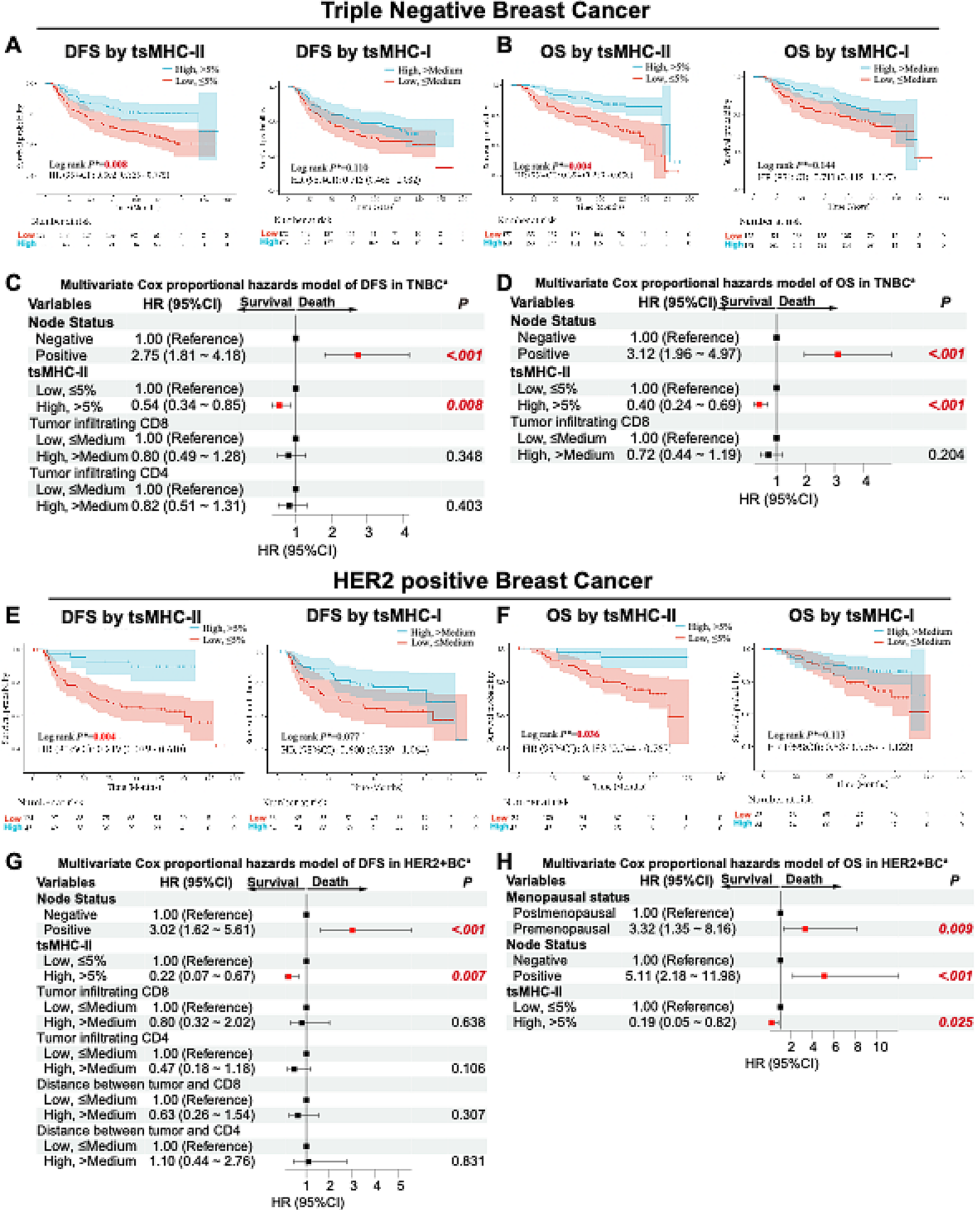
High Expression of tsMHC-II Predicts Better DFS and OS in Breast Cancer. **A**-**B**. Kaplan-Meier survival analysis for DFS (**A**) and OS (**B**) of high vs. low tsMHC-II and tsMHC-I expression groups in TNBC. **C**-**D**. Multivariate Cox regression analysis forest plots for DFS (**C**) and OS (**D**) of high vs. low tsMHC-II and tsMHC-I expression groups in TNBC. **E**-**F**. Kaplan-Meier survival analysis for DFS (**E**) and OS (**F**) of high vs. low tsMHC-II and tsMHC-I expression groups in HER2 + BC. **G**-**H**. Multivariate Cox regression analysis forest plots for DFS (**G**) and OS (**H**) of high vs. low tsMHC-II and tsMHC-I expression groups in HER2 + BC. * indicates P values were corrected by Benjamini-Hochberg method; a indicates variables included in multivariate regression analysis with univariate p values < 0.05

In TNBC, high tsMHC-II (HR = 0.50, 95%CI = 0.33–0.78, *p* = 0.008, Fig. 2A) and CD8 expression (HR = 0.60, 95%CI = 0.40–0.92, *p* = 0.029, Fig. S1A) were associated with better DFS. After multivariate Cox regression adjustment, tsMHC-II high expression remained an independent predictor of better DFS (HR = 0.54, 95%CI = 0.34–0.85, *p* = 0.008, Fig. 2C and S1C). Lymph node metastasis was linked to worse DFS (HR = 2.75, 95%CI = 1.81–4.18, *p* < 0.001, Fig. 2C). For OS, high tsMHC-II (HR = 0.39, 95%CI = 0.24–0.65, *p* = 0.004, Fig. 2B) and CD8 (HR = 0.55, 95%CI = 0.34–0.89, *p* = 0.024, Fig. S1B) predicted better OS. After adjustment, tsMHC-II high expression indicated better OS (HR = 0.40, 95%CI = 0.24–0.69, *p* < 0.001, Fig. 2D and S1D) independently, with lymph node metastasis indicating worse OS (HR = 3.12, 95%CI = 1.96–4.97, *p* < 0.001, Fig. 2D).

In HER2 + BC, high tsMHC-II (HR = 0.22, 95%CI = 0.08–0.61, *p* = 0.004, Fig. 2E), CD4 (HR = 0.41, 95%CI = 0.23–0.75, *p* = 0.006, Fig. 2E), and CD8 (HR = 0.53, 95%CI = 0.30–0.95, *p* = 0.040, Fig. S1E) were associated with better DFS. After multivariate analysis, tsMHC-II high expression (HR = 0.22, 95%CI = 0.07–0.67, *p* = 0.007, Fig. 2G) was the only effective predictor of better DFS, while lymph node metastasis was linked to worse DFS (HR = 3.02, 95%CI = 1.62–5.61, *p* < 0.001, Fig. 2G and S1G). As for OS, high tsMHC-II expression (HR = 0.18, 95%CI = 0.04–0.77, *p* = 0.036, Fig. 2F and S1F) predicted better survival. After adjustment, tsMHC-II high expression (HR = 0.19, 95%CI = 0.05–0.82, *p* = 0.025, Fig. 2H and S1H) remained an independent factor for better OS, while premenopausal status (HR = 3.32, 95%CI = 1.35–8.16, *p* = 0.009, Fig. 2H) and lymph node metastasis (HR = 5.11, 95%CI = 2.18–11.98, *p* < 0.001, Fig. 2H) were associated with worse OS.

### High tsMHC-II expression in TNBC exhibits anthracycline exemption potential

To investigate whether tsMHC-II can guide anthracycline exemption in TNBC, we defined inclusion and exclusion criteria for the anthracycline sequential paclitaxel (EC-P) and paclitaxel with carboplatin (PCb) cohorts. Among 345 TNBC patients, those who had not received NAC and were treated with standard postoperative chemotherapy regimens (EC-P or PCb) were included. Patients receiving non-standard regimens, insufficient doses or cycles, or those with other malignancies or incomplete follow-up were obviated. Eventually, the total of 82 patients with PCb and 198 patients with EC-P were included for subgroup analyzed.

**Fig. 3:**
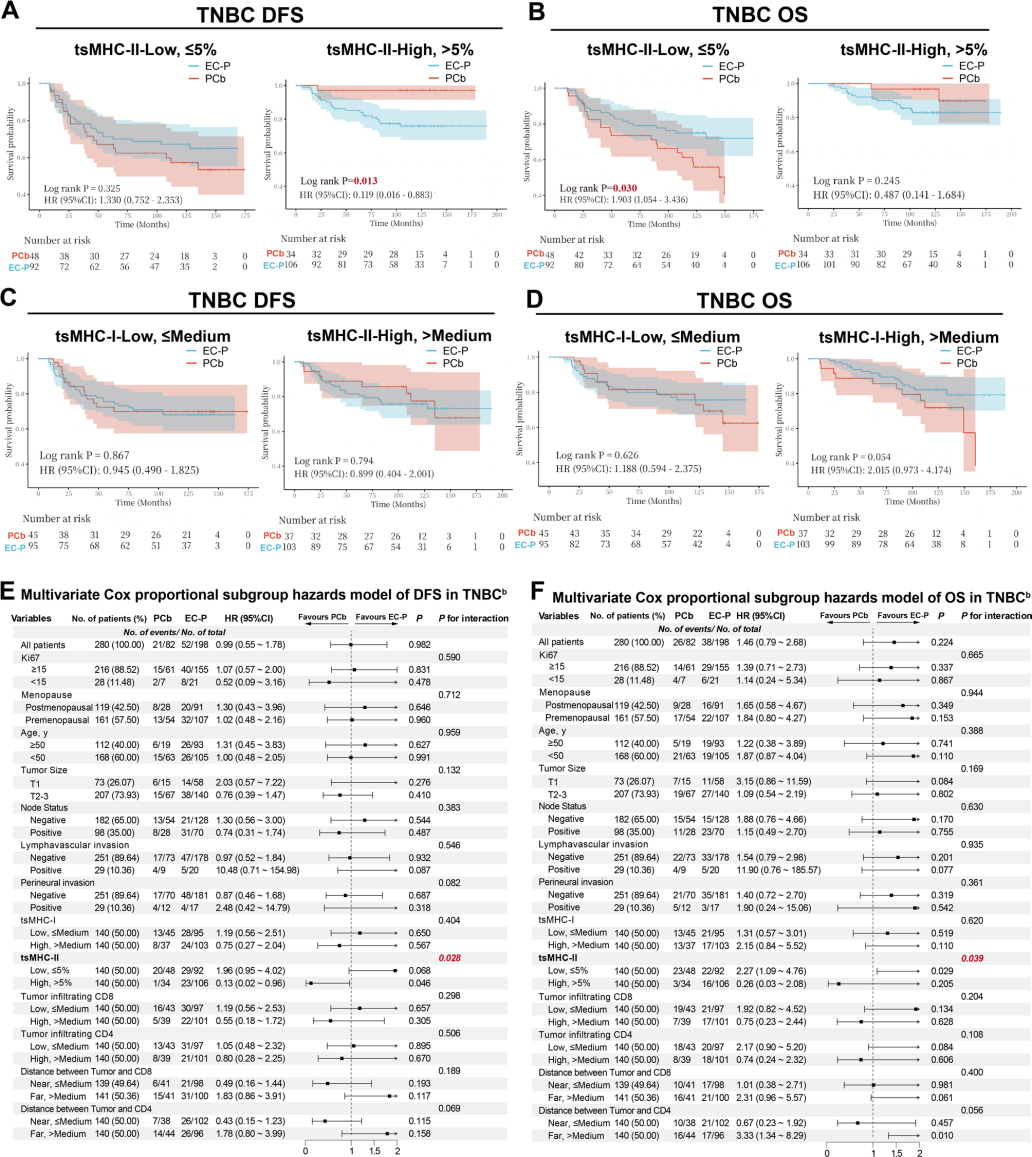
High tsMHC-II Expression in TNBC Exhibits Anthracycline Exemption Potential. A-B. Kaplan-Meier survival analysis of DFS (**A**) and OS (**B**) in TNBC patients with high vs. low tsMHC-II expression under PCb and EC-P chemotherapy regimens. **C**-**D**. Kaplan-Meier survival analysis of DFS (**C**) and OS (**D**) in TNBC patients with high vs. low tsMHC-I expression under PCb and EC-P chemotherapy regimens. **E**-**F**. Multivariate Cox regression analysis forest plots for DFS (**E**) and OS (**F**) comparing the PCb vs. EC-P chemotherapy subgroups in TNBC. b indicates that all factors listed in the table were included as covariates in the multivariate Cox regression analysis

In TNBC, Kaplan-Meier analysis indicated that tsMHC-II high expression derived prominent better DFS from PCb group compared to EC-P group (HR = 0.12, 95%CI = 0.02–0.88, *p* = 0.013, Fig. 3A). In contrast, low tsMHC-II expression showed no DFS discrepancy between PCb and EC-P (HR = 1.33, 95%CI = 0.75–2.35, *p* = 0.325, Fig. 3A). Besides, high or low expression of tsMHC-I, CD4, and CD8 showed no DFS differences between the two regimens (Fig. 3C and S2A and S2C).

To further directly compare the DFS benefit differences between EC-P treatment regimen and PCb treatment regimen among groups with different tsMHC-II expression levels, multivariate Cox regression analysis adjusting for confounding factors confirmed that only tsMHC-II high expression in TNBC predicted significantly better DFS with PCb compared to EC-P (HR = 0.13, 95%CI = 0.02–0.96, P_interaction_=0.028, Fig. 3E).

Kaplan-Meier analysis for OS showed that tsMHC-II low expression patients had better OS with EC-P (HR = 1.90, 95%CI = 1.05–3.44, *p* = 0.030, Fig. 3B), whereas tsMHC-II high expression showed no difference (HR = 0.49, 95%CI = 0.14–1.68, *p* = 0.245, Fig. 3B). Similarly, low CD8 expression showed better OS with EC-P (HR = 2.04, 95%CI = 1.09–3.83, *p* = 0.023, Fig. S2B), while no difference in the high CD8 expression group (HR = 0.91, 95%CI = 0.37–2.20, *p* = 0.831, Fig. S2B). No significant prognostic stratification for OS was observed for tsMHC-I or CD4 between the regimens (Fig. 3D and S2D). After adjustment, it showed that only tsMHC-II low expression significantly benefited from EC-P in OS (HR = 2.27, 95%CI = 1.09–4.76, P_interaction_=0.039, Fig. 3F).

### tsMHC-II exhibits better prediction efficacy of survival benefit in paclitaxel chemotherapy

The previous results have established the potential of tsMHC-II for stratifying TNBC patients for anthracycline exemption. To further analyze its efficacy across chemotherapy regimens, we conducted subgroup analysis of tsMHC-II, tsMHC-I, CD8, and CD4 in the EC-P and PCb subgroups.

**Fig. 4:**
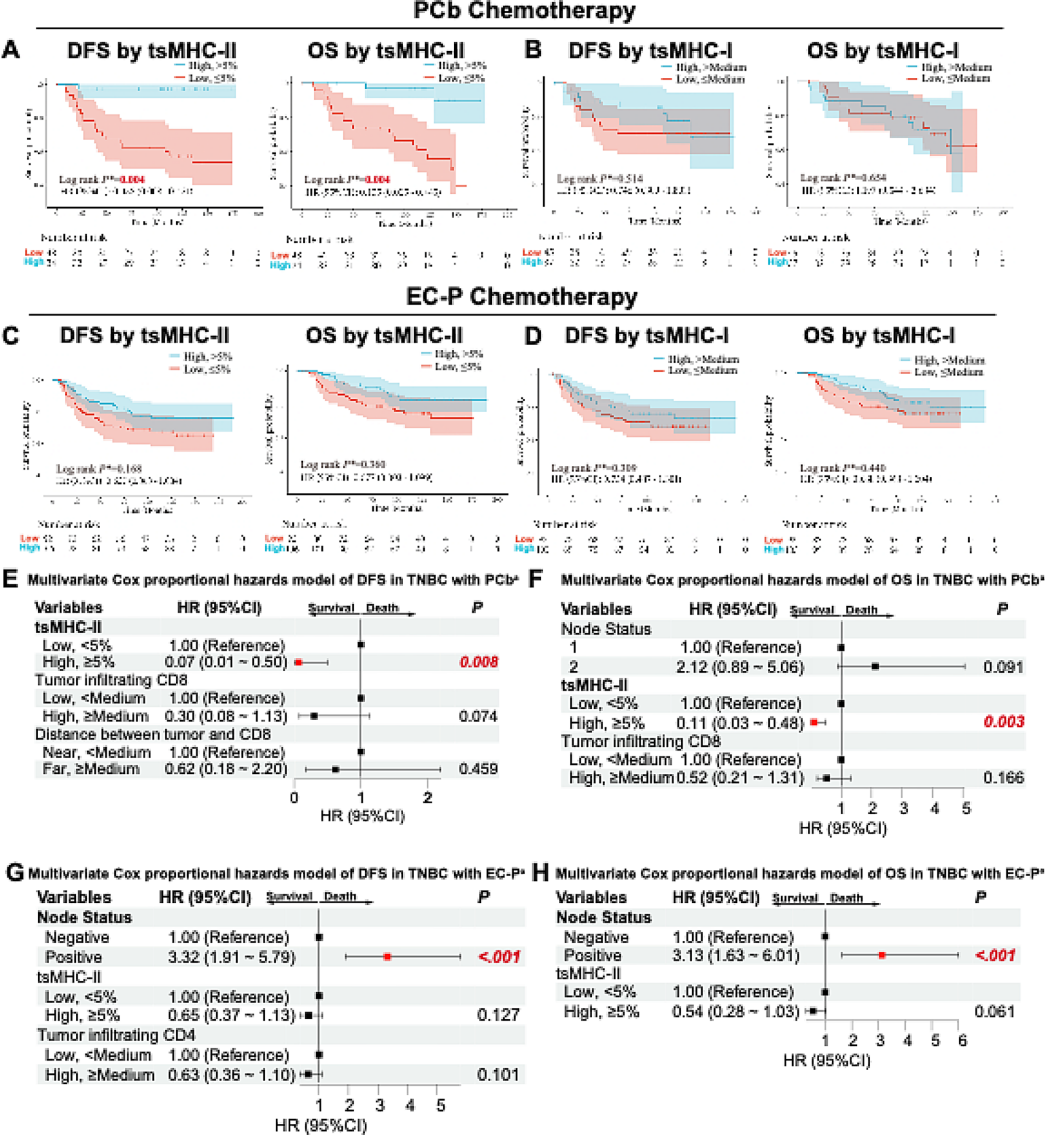
tsMHC-II Exhibits Better Prediction Efficacy of Survival Benefit in Paclitaxel Chemotherapy. A-B. Kaplan-Meier survival analysis of DFS (**A**) and OS (**B**) in TNBC patients receiving PCb chemotherapy, comparing high vs. low tsMHC-II and tsMHC-I expression groups. **C**-**D**. Kaplan-Meier survival analysis of DFS (**C**) and OS (**D**) in TNBC patients receiving EC-P chemotherapy, comparing high vs. low tsMHC-II and tsMHC-I expression groups. **E**-**F**. Multivariate Cox regression analysis forest plots for DFS (**E**) and OS (**F**) in TNBC patients receiving PCb chemotherapy. **G**-**H**. Multivariate Cox regression analysis forest plots for DFS (**G**) and OS (**H**) in TNBC patients receiving EC-P chemotherapy. *P values were corrected using the Benjamini-Hochberg method. a indicates variables included in the multivariate analysis with univariate p values < 0.05

In the PCb subgroup, patients with high tsMHC-II expression had significantly better DFS (HR = 0.06, 95%CI = 0.01–0.43, *p* = 0.004, Fig. 4A) and OS (HR = 0.11, 95%CI = 0.03–0.45, *p* = 0.004, Fig. 4A), as did patients with high CD8 expression (DFS: HR = 0.28, 95%CI = 0.10–0.76, *p* = 0.014; OS: HR = 0.37, 95%CI = 0.15–0.86, *p* = 0.032, Fig. S2E). However, tsMHC-I and CD4 did not stratify DFS or OS in the PCb group (Fig. 4B and S2F).

After multivariate Cox regression adjustment, only tsMHC-II high expression predicted better DFS (HR = 0.07, 95%CI = 0.01–0.50, P_interaction_=0.008, Fig. 4E) and OS (HR = 0.11, 95%CI = 0.03–0.48, *p* = 0.003, Fig. 4F) in TNBC patients receiving PCb chemotherapy (Fig. S3A and S3B).

As for the EC-P subgroup, tsMHC-II, tsMHC-I, CD8, and CD4 did not significantly stratify survival benefits (Fig. 4C, 4D, S2G and S2H). After multivariate Cox regression adjustment, lymph node positive metastasis predicted worse DFS (HR = 3.32, 95%CI = 1.91–5.79, P_interaction_<0.001, Fig. 4G) and OS (HR = 3.13, 95%CI = 1.63–6.01, P_interaction_<0.001, Fig. 4H, Fig. S3C and S3D) in EC-P group TNBC patients. These findings confirm tsMHC-II’s potential in precisely identifying TNBC patients who could benefit from PCb chemotherapy and support the feasibility of anthracycline exemption in tsMHC-II high-expression TNBC patients based on better treatment benefits.

### TNBC validation cohort confirms the efficacy of tsMHC-II in anthracycline exemption stratification

**Fig. 5:**
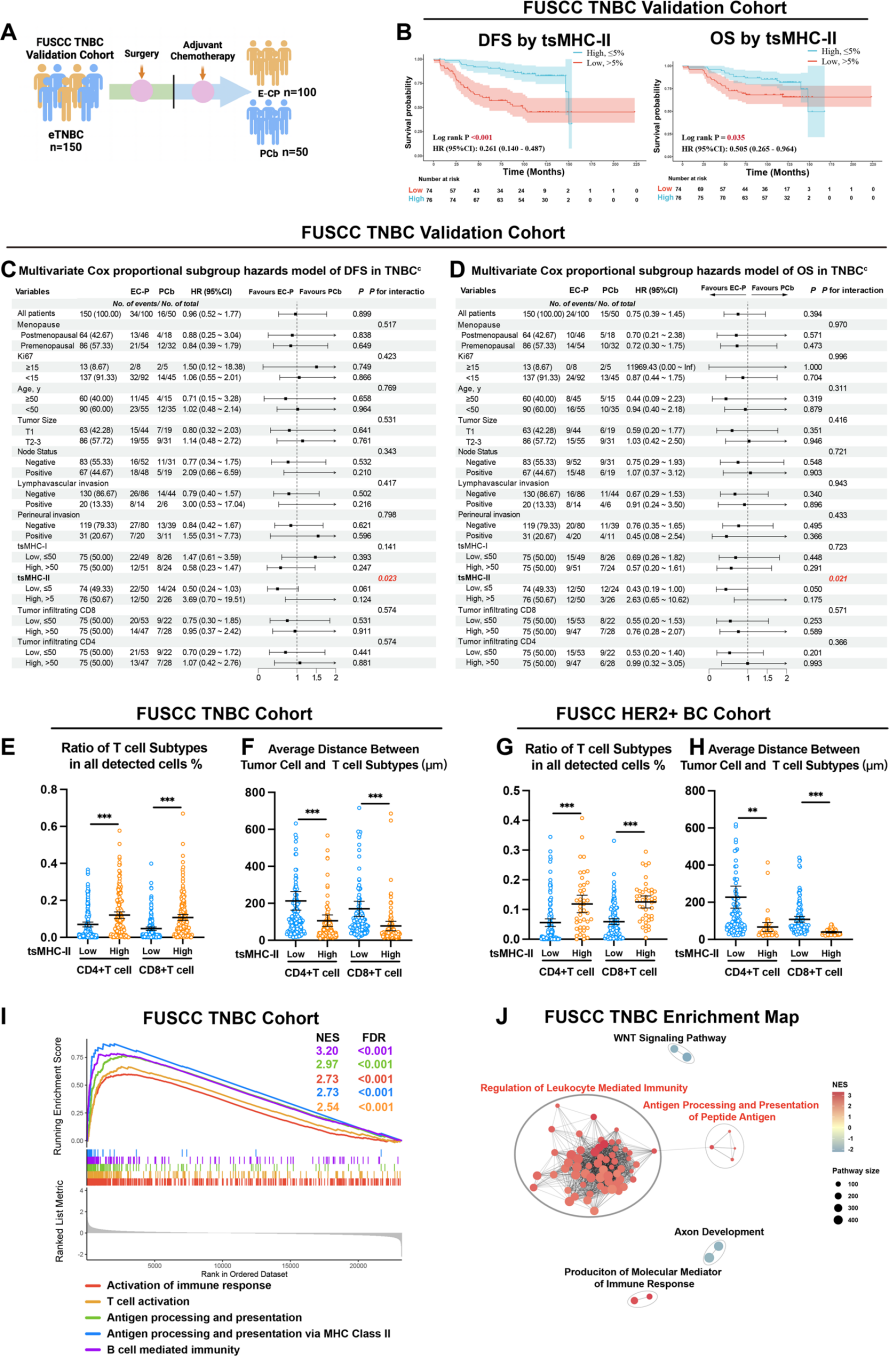
TNBC Validation Cohort Confirms the Efficacy of tsMHC-II in Anthracycline Exemption Stratification. **A**. Schematic diagram of the treatment protocol for the FUSCC TNBC validation cohort. **B**. Survival analysis plots of DFS and OS comparing the tsMHC-II high-expression and low-expression groups in the FUSCC TNBC validation cohort. **C**-**D**. Forest plots of multivariate Cox regression analysis for DFS (**C**) and OS (**D**) comparing the paclitaxel plus carboplatin (PCb) versus anthracycline-sequenced paclitaxel (EC-P) chemotherapy subgroups in the FUSCC TNBC validation cohort. **E**-**F**. Graphs showing differences in the proportions of CD4 + T cells and CD8 + T cells (**E**) and their distance from the tumor (**F**) between the tsMHC-II high-expression and low-expression groups of TNBC patients in the FUSCC exploratory cohort. **G**-**H**. Graphs showing differences in the proportions of CD4 + T cells and CD8 + T cells (**G**) and their distance from the tumor (**H**) between the tsMHC-II high-expression and low-expression groups of HER2 + patients in the FUSCC exploratory cohort. **I**. Upregulated pathways identified by GSEA analysis of paired RNA-seq data from tsMHC-II high-expression patients in the FUSCC exploratory cohort. **J**. Enrichment map displaying upregulated pathways from GSEA analysis of paired RNA-seq data from tsMHC-II high-expression patients in the FUSCC exploratory cohort. *means *p* < 0.05; **means *p* < 0.01; ***means *p* < 0.001; c indicates that variables with univariate *p* < 0.05 were included in the multivariate logistic regression model

To further validate the ability of tsMHC-II to guide anthracycline treatment stratification in TNBC, we concurrently collected data from 150 TNBC patients at FUSCC (Table S1). Of these, 100 received postoperative adjuvant anthracycline combined with taxane therapy, while 50 received taxane-only therapy (A). Using the same mIHC technique as in the previous cohort, we quantified the proportions and positive subgroups of tsMHC-II, tsMHC-I, CD4, and CD8 in these patients. Survival outcomes in the FUSCC-TNBC validation cohort similarly demonstrated that patients with high tsMHC-II expression had significantly better DFS (HR = 0.26, 95% CI = 0.14–0.49, *P* < 0.001, Fig. 5B and S4A) and OS (HR = 0.51, 95% CI = 0.27–0.96, *P* = 0.035, Fig. 5B and S4B) compared to those with low tsMHC-II expression. Multivariate regression analysis revealed that, in the validation cohort, patients with high tsMHC-II expression showed no significant difference in DFS or OS between anthracycline-plus-taxane and taxane-only regimens (Fig. 5C, 5D, S4C and S4D). In contrast, patients with low tsMHC-II expression derived significant DFS and OS benefits from the anthracycline-plus-taxane regimen, with statistically significant intergroup interaction differences, consistent with the findings of the exploratory cohort.

In conclusion, this study found that triple-negative breast cancer patients with high tsMHC-II expression can achieve the same survival benefit from the PCb treatment regimen as from the EC-P treatment regimen, providing a novel therapeutic biomarker for the anthracycline-sparing population in clinical triple-negative breast cancer patients.

### Breast cancer with high tsMHC-II expression exhibits immune cell-rich phenotype and immunotherapy benefit

To characterize the TME associated with tsMHC-II and tsMHC-I in breast cancer, tumors with high tsMHC-II expression demonstrated significantly greater infiltration of CD4 + and CD8 + T cells, along with closer spatial proximity of these T cells to tumor cells (Fig. 5E and 5F). Similar trends were observed in the tsMHC-I high-expression group (Fig. S4E). In HER2 + breast cancer, patients with elevated tsMHC-II expression exhibited enhanced CD8 + and CD4 + T cell infiltration and reduced T cell-to-tumor distances (Fig. 5G and 5H), patterns paralleled in tsMHC-I-high tumors (Fig. S4F).

Gene Set Enrichment Analysis (GSEA) of the matched FUSCC-TNBC cohort RNA-seq datasets revealed that high tsMHC-II expression was associated with significant activation of adaptive immunity pathways, including T/B cell activation and MHC-II-mediated antigen presentation, reflecting robust antitumor immune responses (Fig. 5I and S4G). Enrichment network analysis further confirmed immune-centric pathway activation dominated by immune cell-mediated cytotoxicity and antigen processing (Fig. 5J and S4H).

**Fig. 6:**
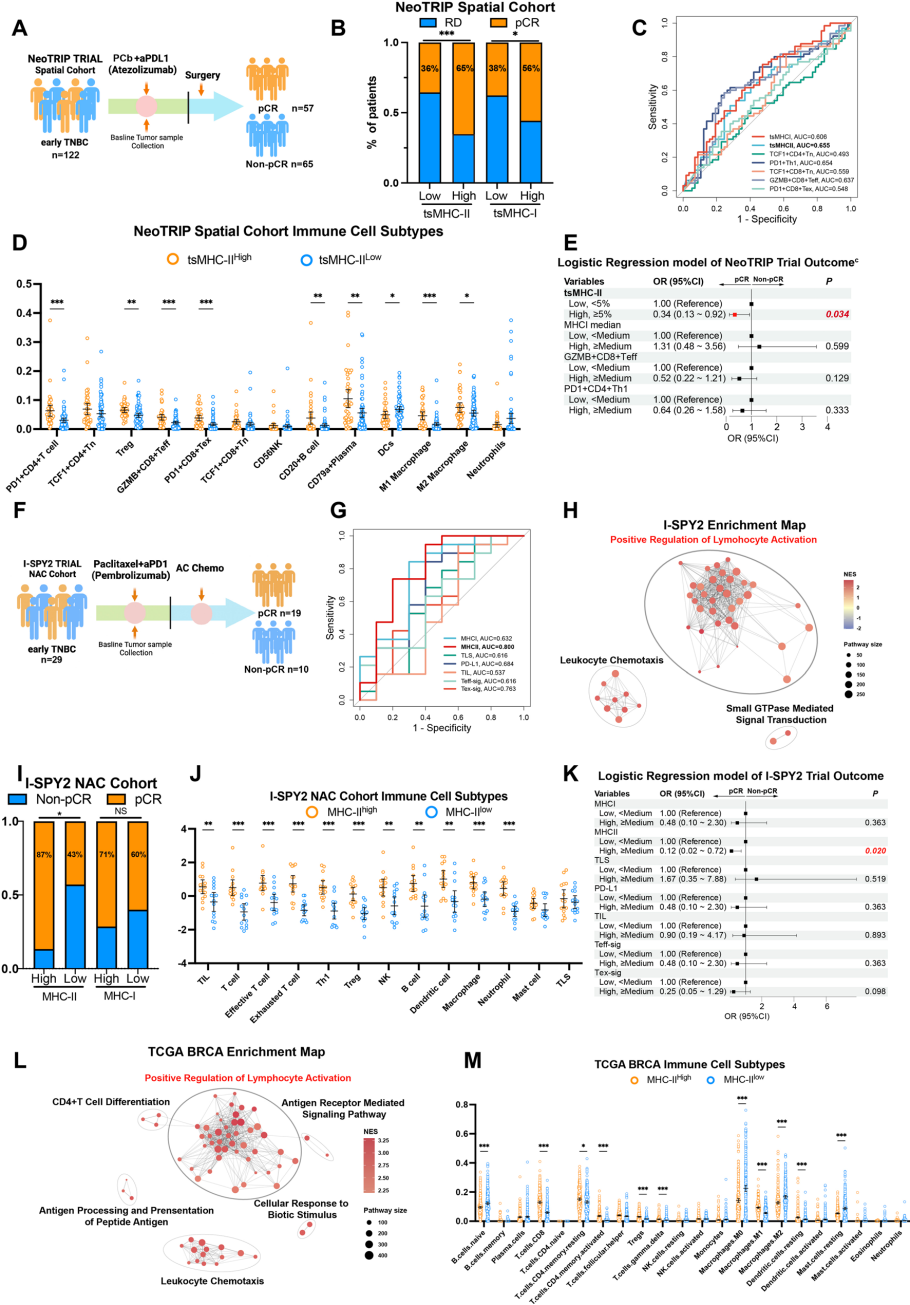
Breast Cancer with High tsMHC-II Expression Exhibits Immune Cell-rich Phenotype and Immunotherapy Benefit. **A** Schematic diagram of the treatment protocol for the NeoTRIP NAC clinical trial spatial cohort. **B** Bar graph comparing the proportions of pathological complete response (pCR) and residual disease (RD) in the tsMHC-II/tsMHC-I high-expression versus low-expression groups in the NeoTRIP cohort, with differences assessed by chi-square test. **C** ROC models predicting treatment outcomes based on tsMHC-II, tsMHC-I, and various T-cell subsets in the NeoTRIP cohort. **D** Graph illustrating differences in the proportions of immune subgroups between the tsMHC-II high-expression and low-expression groups in the NeoTRIP cohort. **E** Forest plot of multivariate logistic regression analysis in the NeoTRIP cohort. **F** Schematic diagram of the treatment protocol for the I-SPY2 TNBC immunotherapy cohort. **G** ROC prediction models for treatment outcomes constructed using transcriptome-based scoring of MHC-I, MHC-II, TLS, PD-L1, TIL, Teff-sig, and Tex-sig pathways in the I-SPY2 TNBC immunotherapy cohort. **H** Enrichment map displaying upregulated pathways from GSEA analysis in the tsMHC-II high-expression group of the I-SPY2 immunotherapy cohort. **I** Bar graph comparing the proportions of pCR and RD in the tsMHC-II/tsMHC-I high-expression versus low-expression groups in the I-SPY2 immunotherapy cohort, with differences assessed by chi-square test. **J** Graph showing differences in immune subgroup scores between the MHC-II pathway high-expression and low-expression groups in the I-SPY2 immunotherapy cohort. **K.** Forest plot of univariate logistic regression analysis in the I-SPY2 immunotherapy cohort. **L.** Enrichment map of upregulated pathways from GSEA analysis in the MHC-II pathway high-expression group of the TCGA BRCA database. **M.** Graph illustrating differences in immune subgroup proportions between the MHC-II pathway high-expression and low-expression groups in the TCGA BRCA database, analyzed using CIBERSORTx. *means *p* < 0.05; **means *p* < 0.01; ***means *p* < 0.001; c indicates that variables with univariate *p* < 0.05 were included in the multivariate logistic regression model

In the NeoTRIP trial evaluating neoadjuvant PCb chemotherapy combined with Atezolizumab ( n = 122), tumors with high tsMHC-II expression showed significantly higher pathological complete response (pCR) rates compared to low-expression counterparts (Fig. 6A and 6B). An outcome-predictive ROC model demonstrated that tsMHC-II surpassed tsMHC-I, PD1 + Th1 cells, PD1 + CD8 + exhausted T cells (Tex), and GZMB + CD8 + effector T cells (Teff) in predictive accuracy (Fig. 6C). Spatial proteomics revealed increased infiltration of PD1 + CD4 + Th1 cells, GZMB + CD8 + Teff cells, PD1 + CD8 + Tex cells, CD20 +B cells, plasma cells, dendritic cells, and M1 macrophages in tsMHC-II-high tumors (Fig. 6D). Multivariate analysis identified tsMHC-II-high status as an independent predictor of improved treatment response (HR = 0.34, 95%CI = 0.13–0.92, *p* = 0.034; Fig. 6E and. S5A).

Validation using I-SPY2 trial data (paclitaxel + immunotherapy arm) confirmed that TNBC patients with high MHC-II pathway scores achieved significantly higher pCR rates compared to MHC-I-high counterparts (Fig. 6F and 6I). ROC analysis demonstrated MHC-II’s superior predictive performance over tertiary lymphoid structures (TLS), TILs, PD-L1, Tex-sig, and Teff-sig (Fig. 6G). GSEA highlighted immune chemotaxis and activation pathway enrichment in MHC-II-high tumors (Fig. 6H and S5B), while immune subset analysis revealed elevated antitumor effector T cells, B cells, and macrophages (Fig. 6J). High MHC-II expression correlated with improved NAC-IT outcomes (OR = 0.12, 95%CI = 0.02–0.72, *p* = 0.02; Fig. 6K).

Analysis of TCGA breast cancer data further showed that high MHC-II expression predicted prolonged recurrence-free survival (RFS) and OS (Fig. S5C and S5D). Transcriptomic profiling confirmed enrichment of CD4 + T cell differentiation and antigen presentation pathways in tsMHC-II-high tumors (Fig. 6L, S5E and S5F), accompanied by increased T/B cells and M1 macrophages (Fig. 6M).

Collectively, multi-cohort validation including FUSCC-TNBC, NeoTRIP, I-SPY2, TCGA established that tsMHC-II-high TNBC tumors exhibit: (1) immune-hot phenotypes characterized by spatial immune infiltration, (2) adaptive immunity pathway activation, and (3) superior predictive value for immunotherapy response compared to TILs and PD-L1.

In conclusion, TNBC patients with high tsMHC-II expression demonstrate superior anti-tumor immune efficacy, and tsMHC-II can serve as a biomarker for selecting high-efficacy benefiting populations for NAC-IT in TNBC patients.

### KAT2B regulates tsMHC-II function as a dual-effect biomarker for anthracycline chemotherapy exemption and immunotherapy benefit

**Fig. 7:**
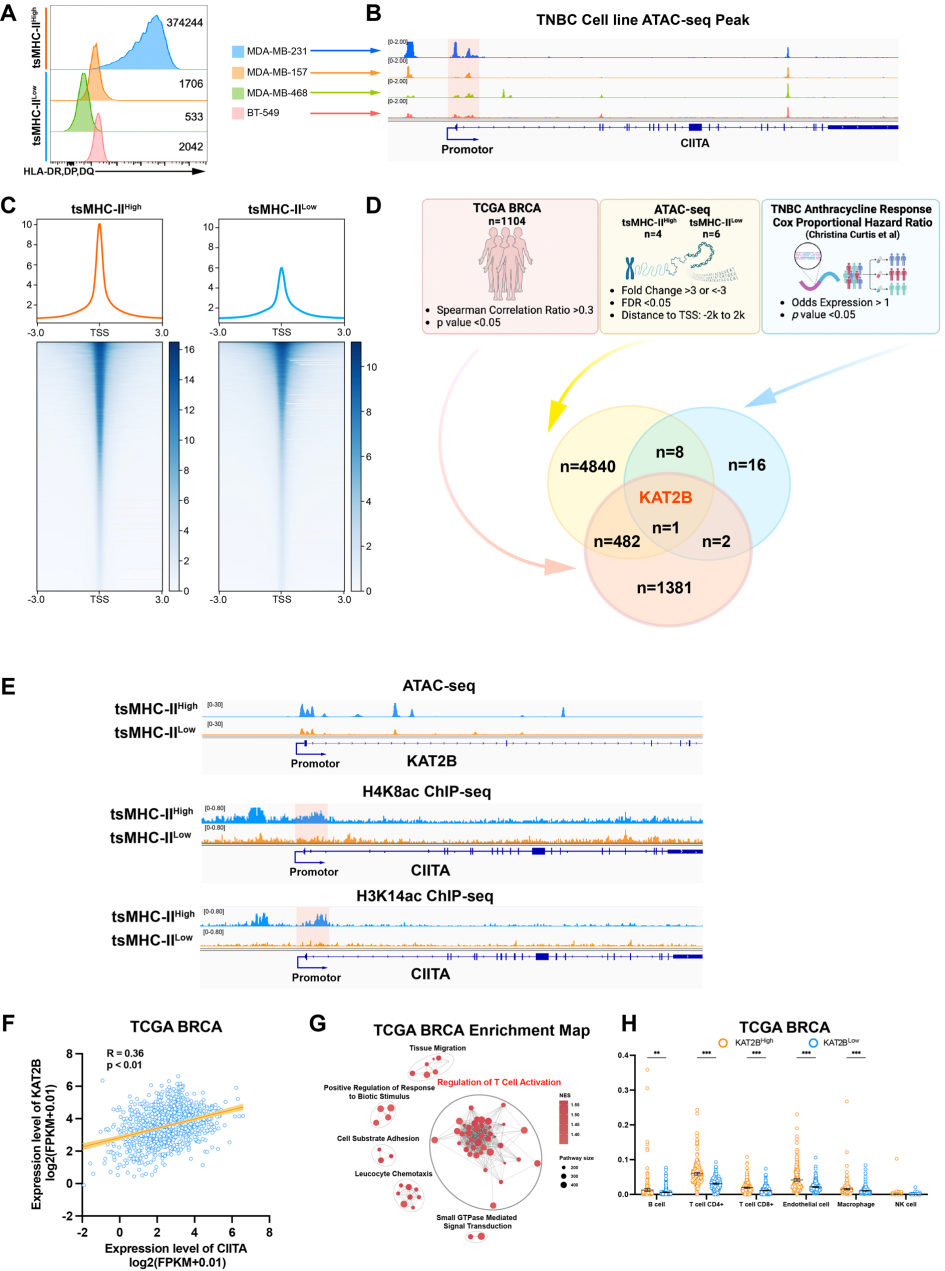
KAT2B Regulates tsMHC-II Function as a Dual-Effect Biomarker for Anthracycline Chemotherapy Exemption and Immunotherapy Benefit. **A** Flow cytometry peak plots showing the protein expression levels of HLA-DR, DQ, and DP in TNBC cell lines (MDA-MB-231, BT-549, MDA-MB-468, MDA-MB-157). **B** ATAC-seq peak plots of the CIITA gene in TNBC cell lines (MDA-MB-231, BT-549, MDA-MB-468, MDA-MB-157), with the promoter region highlighted in red shading. **C** ATAC-seq peak plots near transcription start sites (TSS) comparing TNBC cell lines with high versus low tsMHC-II expression. **D** Schematic diagram and Venn diagram illustrating the identification of KAT2B as a regulatory gene for tsMHC-II, screened by integrating TCGA database, TNBC cell line ATAC-seq data, and the Curtis’s anthracycline chromatin therapeutic efficacy-related database. **E** ATAC-seq peak plots of KAT2B and ChIP-seq peak plots of H4K8ac and H3K14ac at the CIITA gene in TNBC cell lines with high versus low tsMHC-II expression, with the CIITA promoter region highlighted in red. **F** Pearson correlation scatter plot of CIITA and KAT2B expression levels in the TCGA BRCA database. **G** Enrichment map of upregulated pathways in the KAT2B high-expression group from GSEA analysis of the TCGA BRCA database. **H** Bar graph showing differences in cell subpopulation proportions between KAT2B high- and low-expression groups in the TCGA BRCA database, analyzed using EPIC

Chromatin regulatory elements influence the efficacy of anthracycline therapy and immunotherapy benefits in breast cancer. To further explore the mechanism by which tsMHC-II exerts its dual-effect functionality as a predictive biomarker for both anthracycline exemption and immunotherapy benefit, we first characterized tsMHC-II expression levels across TNBC cell lines. Based on expression profiles, we stratified these cell lines into high tsMHC-II expression (MDA-MB-231) and low tsMHC-II expression (MDA-MB-468, MDA-MB-157, BT-549) groups (Fig. 7A and S6A). ATAC-seq profiling revealed significantly elevated chromatin accessibility at the CIITA promoter, a key transcriptional regulator of tsMHC-II, with globally increased chromatin openness in MDA-MB-231 relative to low tsMHC-II cell lines, suggesting that tsMHC-II high-expressing TNBC exhibits enhanced chromatin epigenetic regulatory activity (Fig. 7B and 7C). Concurrently, high tsMHC-II-expressing tumor cells exhibited a closed chromatin state at the ABCB1 promoter and an open state at TOP2A (Fig. S6B).

To further investigate the epigenetic mechanisms of tsMHC-II upregulation, we integrated: (1) TCGA data showing a Spearman correlation > 0.3 (*p* < 0.05) between CIITA and gene expression; (2) differential ATAC-seq analysis between tsMHC-II-high (*n* = 4; MDA-MB-231) and tsMHC-II-low (*n* = 6; MDA-MB-468, MDA-MB-157, BT-549) cell lines, identifying genes with significantly differential promoter accessibility; and (3) Curtis et al.’s epigenetic gene set linked to anthracycline therapy correlation in TNBC [39]. This multi-omics convergence identified KAT2B as a putative tsMHC-II modulator (Fig. 7D and Table S3). TCGA analysis confirmed significant CIITA-KAT2B co-expression (Fig. 7E). ATAC-seq confirmed increased chromatin accessibility in the promoter and exon regions of KAT2B in tsMHC-II high-expressing TNBC cell lines relative to tsMHC-II low-expressing cell lines. Simultaneously, ChIP-seq demonstrated heightened H4K8/H3K14 acetylation, which are hallmarks of KAT2B regulatory marks, at the CIITA promoter in tsMHC-II-high compared with tsMHC-II-low cells (Fig. 7E). GSEA of TCGA cohorts revealed a significant high correlation between KAT2B and CIITA RNA expression levels in breast cancer. Additionally, KAT2B-high tumors were enriched for T-cell activation and immune chemotaxis pathways, with immune deconvolution showing elevated antitumor T/B cells and macrophages (Fig. 7F, 7G, 7H, S6C and S6D). Drug sensitivity analysis indicated KAT2B-high patients exhibited greater paclitaxel sensitivity without altered anthracycline response (Fig. S6G and S6H). Clinically, high KAT2B expression correlated with improved RFS and OS in TNBC and HER2 + BC (Fig. S6E and S6F).

These findings confirm that tsMHC-II high-expressing TNBC exhibits enhanced chromatin accessibility, especially CIITA, the core regulatory transcription factor of tsMHC-II, and epigenetic regulatory activity, which constitutes a critical feature enabling its predictive role in anthracycline exemption and immunotherapy benefit assessment. Furthermore, KAT2B emerges as a potentially crucial epigenetic modifier that regulates tsMHC-II upregulation through CIITA.

### KAT2B acetylation of CIITA promoter upregulates tumor-specific MHC-II expression enhancing NAC-IT efficacy

**Fig. 8:**
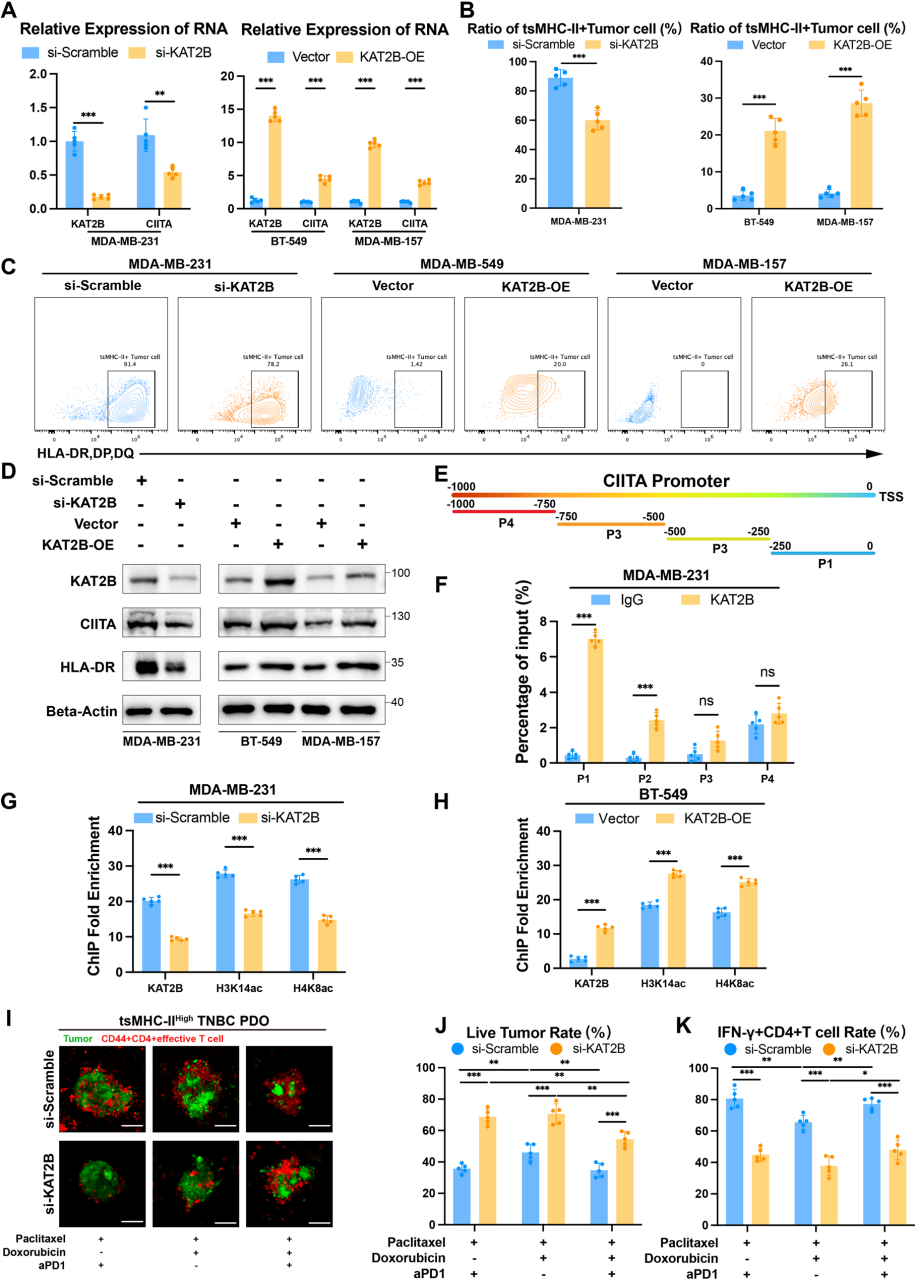
KAT2B Acetylation of CIITA Promoter Upregulates Tumor-Specific MHC-II Expression Enhancing NAC-IT Efficacy. **A** Relative RNA expression of KAT2B and CIITA in MDA-MB-231 cells with KAT2B knockdown versus control group (Left); Relative RNA expression of KAT2B and CIITA in BT-549 and MDA-MD-157 cells with KAT2B overexpression versus control group (Right). **B** Flow cytometry analysis showing proportion of tsMHC-II positive tumor cells in MDA-MB-231 with KAT2B knockdown versus control group (Left); Proportion of tsMHC-II positive tumor cells in BT-549 and MDA-MD-157 with KAT2B overexpression versus control group (Right). **C.**Radar plots of flow cytometry data for MDA-MB-231 with KAT2B knockdown, BT-549 and MDA-MD-157 with KAT2B overexpression compared to their respective control groups. **D.**Western Blot analysis of KAT2B, CIITA, HLA-DR, and Beta-Actin protein expression in MDA-MB-231 with KAT2B knockdown, BT-549 and MDA-MD-157 with KAT2B overexpression compared to their respective control groups. **E.** Schematic diagram of ChIP-qPCR primer design for the 1000 bp CIITA promoter fragments. **F.** ChIP-qPCR bar graph showing KAT2B binding enrichment at CIITA promoter fragments P1, P2, P3, and P4. **G-H.** ChIP-qPCR bar graphs showing enrichment of KAT2B, H3K14ac, and H4K8ac at the CIITA promoter P1 segment in MDA-MB-231 with KAT2B knockdown, BT-549 and MDA-MD-157 with KAT2B overexpression compared to their respective control groups. **I.** Fluorescence imaging results of co-culture between tsMHC-II high-expression TNBC PDOs and paired CD44 + CD4 + effector T cells. Red indicates CD44 + CD4 + effector T cells, and green represents tsMHC-II high-expression TNBC PDOs. Scale bar: 50 μm. **J-K.** Tumor viability (**J**) and IFN-γ + CD4 + T-cell proportion (**K**) in PDO immune co-culture groups treated with paclitaxel, doxorubicin, or aPD1, respectively. *means *p* < 0.05; **means *p* < 0.01; ***means *p* < 0.001

To further elucidate KAT2B’s regulation of CIITA, we knocked down KAT2B in tsMHC-II-high MDA-MB-231 cells, resulting in significantly reduced CIITA RNA expression, while overexpression in tsMHC-II-low BT-549 and MDA-MB-157 cells significantly increased CIITA RNA levels (Fig. 8A). Flow cytometry confirmed KAT2B knockdown reduced tsMHC-II protein in MDA-MB-231, whereas KAT2B overexpression elevated tsMHC-II protein in BT-549 and MDA-MB-157 (Fig. 8B and 8C). Western blot verified KAT2B mediates upregulation of both CIITA and HLA-DR protein (Fig. 8D).Our ATAC-seq data previously demonstrated that the CIITA promoter region was significantly more accessible in tsMHC-II high-expressing TNBC. To further investigate whether KAT2B mediated histone acetylation in the CIITA promoter region, we divided the 1000 bp CIITA promoter into four consecutive 250 bp fragments (P1, P2, P3, P4). ChIP-qPCR using KAT2B antibody revealed that the P1 segment was the primary KAT2B-enriched region of the CIITA promoter (Fig. 8E and 8F). To further validate KAT2B’s regulatory role in P1 segment acetylation, we performed ChIP-qPCR in KAT2B-knockdown versus control MDA-MB-231 cells, revealing significantly reduced H3K14ac and H4K8ac enrichment at the CIITA promoter P1 segment following KAT2B knockdown. Similarly, ChIP-qPCR in KAT2B-overexpressing versus control BT-549 cells showed significantly increased H3K14ac and H4K8ac enrichment at the CIITA promoter P1 segment (Fig. 8G and 8H). These results confirmed that KAT2B promoted transcriptional activation through acetylation of the CIITA promoter region.

Finally, we sought to explore whether KAT2B directly influences the efficacy of NAC-IT. Functional validation using patient-derived organoids (PDOs) co-cultured with autologous CD44 + CD4 + effector T cells demonstrated equivalent antitumor efficacy between paclitaxel + immunotherapy and anthracycline-paclitaxel + immunotherapy in tsMHC-II-high PDOs, with comparable T-cell activation (Fig. 8I). Meanwhile there was a superior activity of paclitaxel + immunotherapy over anthracycline-containing regimens. Moreover, KAT2B knockdown abolished tsMHC-II expression, reversing therapeutic hierarchy with anthracycline-paclitaxel + immunotherapy outperforming paclitaxel-based therapy (Fig. 8J and8K).

These findings established that KAT2B sustained tsMHC-II expression via CIITA promoter acetylation, driving chromatin accessibility. The PDO-immune cell co-culture system validated that tsMHC-II-high tumors achieve optimal NAC-IT benefits with KAT2B-dependent, anthracycline-sparing regimens, highlighting KAT2B’s pivotal role in maintaining tsMHC-II-mediated immunotherapeutic vulnerability.

## Discussion

This study, for the first time, identified through mIHC combined with a breast cancer tissue microarray cohort that tsMHC-II can serve as a marker for anthracycline exemption in postoperative TNBC patients. Additionally, survival prognostic studies and TME characteristic studies of patients with high tsMHC-II expression in HER2-positive breast cancer remain unexplored. This study found that high tsMHC-II expression in both TNBC and HER2 + BC is correlated with better DFS and OS, making it a robust prognostic marker across breast cancer subtypes, confirming tsMHC-II as a potential prognostic predictor across breast cancer subtypes.Additionally, high tsMHC-II expression serves as an effective predictive indicator for immunotherapy benefit, with tumors exhibiting high expression characterized by the presence of antitumor effector subsets such as T cells, B cells, and macrophages, alongside pathway features dominated by immune cell activation and antigen presentation. This provides a strong biological foundation for tsMHC-II high-expressing TNBC patients to benefit from anthracycline exemption and immunotherapy.

The question of whether certain TNBC patients can be exempt from anthracycline-based chemotherapy during both adjuvant and neoadjuvant stages has been a key research focus. The DBCG 07-READ Trial, with a 10-year follow-up, found that sequential anthracycline and paclitaxel chemotherapy provided better distant disease-free survival (DDFS) and DFS compared to paclitaxel therapy, though OS showed no significant difference, suggesting a conflict between anthracycline-induced heart failure and its long-term tumor benefits [11]. The trial also found that TOP2A amplification led to better DDFS and DFS, but did not affect OS, indicating that TOP2A is not a sufficient marker for anthracycline exemption [39]. The EBCTCG meta-analysis of 18,103 breast cancer patients showed that sequential anthracycline and paclitaxel therapy did not significantly reduce recurrence risk compared to paclitaxel alone, but anthracycline plus taxane regimens significantly reduced recurrence risk and mortality [1]. The PATTERN Trial confirmed that the PCb regimen significantly improved DFS over ECF-T in TNBC patients, with no difference in OS [10]. In our study of TNBC exploration cohort, using mIHC, we found that tsMHC-II high expression had no difference of OS between two chemotherapy regimens, but significantly better DFS with paclitaxel monotherapy. In contrast, tsMHC-II low expression patients showed significant OS benefit from sequential anthracycline and paclitaxel chemotherapy, with no difference in DFS. Concurrently, we validated the aforementioned conclusions using a cohort of 150 TNBC validation cohort. These findings suggested that tsMHC-II high expression might allow TNBC patients to be exempt from anthracycline therapy while still benefiting from DFS, making tsMHC-II a promising biomarker for selecting patients who could avoid anthracycline-based treatment.

MHC-II traditionally expressed on antigen-presenting cells like macrophages, dendritic cells, and B cells, is also found on neutrophils with antigen-presenting functions [40–43]. TsMHC-II has been linked to better prognosis across various cancers and improved outcomes with immune checkpoint inhibitors in melanoma [25, 44, 45]. In breast cancer, tsMHC-II activates CD4 + T cells by presenting tumor-specific neoantigens, distinct from tsMHC-I, driving anti-tumor immunity [29]. In TNBC, high tsMHC-II expression correlates with more tumor-infiltrating lymphocytes (TILs) and activation of the interferon-gamma pathway [24]. While some studies in TNBC have found that HLA-DR high expression is associated with better DFS [28], high-quality research cohorts with tumor region segmentation and reliable OS data have been lacking, particularly in HER2 + BC [38, 46]. This study, using mIHC, quantifies tsMHC-II expression at the protein level and compares it with tsMHC-I, CD4, and CD8. We found that high tsMHC-II expression in TNBC and HER2 + BC patients indicates better DFS and OS, suggesting tsMHC-II as a powerful prognostic marker for adjuvant chemotherapy. Meanwhile, we simultaneously obtained the same conclusion using the TCGA database.

Spatial distance analysis revealed that high tsMHC-II expression leads to closer tumor proximity to CD4 + and CD8 + T cells, indicating stronger anti-tumor potential. In addition, we corroborated these findings through multiple datasets, including FUSCC paired RNA-seq data from TNBC mIHC patients, the NeoTRIP spatial cohort, the I-SPY2 cohort, and TCGA data, at both the spatial proteomic and transcriptomic levels. These analyses confirmed that TNBC with high tsMHC-II expression exhibited a tumor microenvironment characterized by significant enrichment of effector T cells, B cells, plasma cells, DCs, and M1 macrophages, with dominant pathways of immune cell activation, chemotaxis, and antigen presentation. These results provided a mechanistic basis supporting that patients with high tsMHC-II expression could be exempt from anthracycline-based therapy and derive greater benefit from immunotherapy.

The widespread use of NAC-IT in TNBC has improved pCR and EFS outcomes. While patients with high tsMHC-II expression exhibit enhanced immune cell infiltration, providing a cornerstone for immunotherapy response. Retrospective studies show that tsMHC-II expression > 5% in HR + and TNBC patients significantly predicts benefit from durvalumab NAC and Pembrolizumab NAC regimens, highlighting tsMHC-II as a strong prognostic marker for NAC-IT [33, 38]. This trend is only present in the NAC-IT arms, not in chemotherapy-only arms, confirming the potential for tsMHC-II high expression to predict immunotherapy benefit. This study, by integrating MHC-II scoring from the I-SPY2 immunotherapy cohort, performed a cross-sectional comparison with TILs, PD-L1, TLS, and T-cell pathways, revealing that tsMHC-II exhibits the highest predictive efficacy for immunotherapy benefit.The NeoTRIP spatial proteomics study confirmed that MHC-I^+^MHC-II^+^ tumor cell expression is the most relevant predictor of pCR in anthracycline-free PCb regimens [34]. However, the NeoTRIP Spatial research did not integrate comparisons between tumor and microenvironmental immune cells regarding immunotherapy-benefit efficacy. Through a cross-sectional comparison of the predictive efficacy of tsMHC-II with exhausted and effector T-cell subsets, we found that tsMHC-II demonstrates the highest predictive efficacy for outcomes in NAC-IT.

Previous studies have revealed the regulatory mechanisms of tsMHC-II at the epigenetic level. In melanoma, the PRC2/EZH2 pathway mediates H3K27 hypermethylation, suppressing tsMHC-II expression and ultimately leading to immunotherapy resistance [47]. In B-cell lymphoma, PRC1 upregulation silences H2K119Ub, inhibiting tsMHC-II upregulation and contributing to immunotherapy resistance [48]. Additionally, tsMHC-II-presented tumor neoantigens have more stable mutations, suggesting that tsMHC-II and CD4 + T cells play a more deep-seated role in anti-tumor immune surveillance than previously thought [30]. Through a comprehensive analysis integrating CIITA transcriptome expression correlation, chromatin accessibility features of genes in tsMHC-II high-expression tumor cells, and anthracycline treatment-related datasets, we identified that KAT2B induces chromatin accessibility and expression of the CIITA promoter via acetylation. In vitro knockdown experiments combined with PDO immune co-culture assays further confirmed that KAT2B was critical for maintaining high tsMHC-II expression and deriving benefits from immunotherapy. KAT2B, a histone acetyltransferase (HAT), regulates gene expression and protein function by catalyzing lysine residue acetylation. It acetylates histones such as H3K9 and H3K14 to relax chromatin structure and enhance transcription factor binding, while also modifying non-histone substrates to modulate their activity, stability, and localization. KAT2B exhibits tumor-suppressive roles in cervical, cholangiocarcinoma, and ovarian cancers [49, 50]. Notably, KAT2B co-acetylates PLK4 with KAT2A at K45/K46 sites, suppressing its kinase activity to prevent centrosome amplification leading to cancer-associated chromosomal instability [51]. This study first identified that KAT2B transcriptionally upregulates tsMHC-II via CIITA to potentiate immunotherapy. Targeting KAT2B with specific agonists may offer novel therapeutic strategies to enhance immunotherapy efficacy in immune-cold TNBC.

This single-center retrospective study, involving 662 breast cancer patients, has limitations, such as constraints in the inclusion criteria, regional biases in patient selection, time-specific treatment regimens, and insufficient deeper exploration of the immune activation mechanisms of tsMHC-II. This study primarily included East Asian populations, predominantly Chinese individuals. Previous research has confirmed that tsMHC-II expression varies among different ethnic groups [38]. Whether these differences impact tsMHC-II detection and the efficacy of biomarker requires further verification through the inclusion of more diverse patient populations. Despite the presence of a single-center validation cohort, the absence of a multi-center retrospective validation cohort remains a limitation. The conclusions of this study urgently necessitate the inclusion of patients from multiple centers with survival follow-up data. Additionally, the cutoff value for tsMHC-II expression requires further validation. While 5% is commonly used as the threshold for tsMHC-II high expression, further prospective studies are needed to validate the optimal cutoff.

The Department of Breast Surgery at Fudan University Shanghai Cancer Center is planning prospective trials to assess whether tsMHC-II can guide anthracycline exemption in postoperative TNBC chemotherapy and identify patients likely to benefit from NAC-IT. Early-stage TNBC patients with high tsMHC-II expression will be included in anthracycline-free PCb chemotherapy or EC-P chemotherapy groups for non-inferiority survival analysis on DFS, OS, and grade 3 or higher adverse events, with tsMHC-II low expression patients receiving EC-P as a control group. These trials, combined with spatial transcriptomics, will explore the mechanisms of tsMHC-II-mediated immune responses, and identify potential therapeutic targets for tsMHC-II low-expressing patients.

## Conclusions

This study identified tsMHC-II as a dual-efficacy biomarker in operable TNBC patients, guiding anthracycline-free chemotherapy and immunotherapy benefit. High tsMHC-II TNBC patients receiving adjuvant therapy achieve equivalent OS with anthracycline-free paclitaxel-carboplatin regimens, offering a strategy to precisely de-escalate chemotherapy and spare selected patients from anthracycline-related toxicities without compromising outcomes. Meanwhile, high tsMHC-II TNBC patients undergoing neoadjuvant chemotherapy showed enhanced immunotherapy response, with predictive efficacy superior to CPS, PD-L1, TILs, TLS, and T cell exhaustion signatures. High tsMHC-II TNBC displayed core features of enhanced immune cell activation, chemotaxis and infiltration, with KAT2B acetylation of CIITA promoter regions mediating tumor cell tsMHC-II upregulation as a potential mechanism, suggesting novel therapeutic targets for enhancing tsMHC-II expression and immunotherapy response in tsMHC-II-low tumors. Moreover, in both TNBC and HER2-positive breast cancer, high tsMHC-II expression indicated improved DFS and OS.

## Materials and methods

### Study approval and patient tissues

This study involves 662 cases (FUSCC exploration cohort and validation cohort) of 11 sets of 5 mm thick, untreated breast cancer tissue microarray paraffin samples, all obtained from the Breast Surgery Department at Fudan University Shanghai Cancer Center between 2007 and 2014. All patients signed informed consent for the use of their specimens. This study included East Asian women aged 25–70 years with no attrition, and random grouping was applied based on inclusion criteria. The molecular subtypes of the tumors, including TNBC and HER2 + BC, were independently evaluated and determined by the Department of Pathology at Fudan University Shanghai Cancer Center based on ER, PR, and HER2 status. After that, a total of 495 triple-negative breast cancer patients who qualified for either EC-P or PCb regimens were divided into an exploration cohort of 345 patients and a validation cohort of 150 patients at a 2:1 ratio. Both groups had identical inclusion timeframes and criteria. Detailed clinical information matching for the patients is provided in the cohort characteristics (Table S1).

### mIHC

After deparaffinization of FFPE TMA 4 mm samples, antigen retrieval was performed using pH 6 citrate buffer. Staining followed the Absin S7-plex Multiplex Fluorescent Immunohistochemistry Kit protocol (abs50015, Absin). Briefly, samples were treated with 3% hydrogen peroxide, followed by goat serum (SeriveBio) blocking for 15 min. Primary antibodies were incubated for 1 h, then incubated with HRP-conjugated secondary antibodies (Invitrogen) for 30 min. After PBS washing, samples were stained with TSA fluorescent dyes for 15 min. Confocal microscope (Leica) confirmed proper staining in positive and negative controls. A second cycle of staining was started using pH 6 citrate buffer antigen retrieval. After cycles of staining finish, samples were incubated with DAPI for nuclear staining and mounted with anti-fade medium (Panovue). Human tonsil and mouse spleen samples served as positive and negative controls, respectively. All the antibodies used in this study were as follows: tsMHC-II: Monoclonal Mouse Anti-Human HLA-DR/DP/DQ/DX Antibody (Clone.CR3/43, 1:1000, sc-53302, Santa-Cruz); PanCK: Monoclonal Mouse Anti-Human Cytokeratin Antibody (Clone.AE1/AE3, GA053, 1:100, Dako); tsMHC-I: Monoclonal Mouse Anti-HLA Class 1 ABC Antibody (Clone.EMR8-5, ab70328, 1:200, Abcam); CD4: Monoclonal Mouse Anti-Human CD4 Antibody (Clone.4B12, IR649, 1:100, Dako); CD8: Monoclonal Mouse Anti-Human CD8 Antibody (Clone.C8/144B, IR623, 1:150, Dako). The protocol is based on previous research [52].

### Spatial quantification and analysis

Fluorescent signals of TMA were scanned with the PhenoImager HT (Akoya) and analyzed using Qupath v0.5.1 software for cell segmentation, annotation, and spatial distance analysis. Cell segmentation was performed using DAPI, with PanCK serving as a marker to distinguish tumor epithelial from non-tumor structures. Non-analyzable regions, including in situ breast carcinoma, necrotic tissue, normal breast tissue, tissue folds, and non-specific staining, were reviewed and excluded by two pathologists from the central pathology department in a blinded manner. Areas with faint PanCK staining were manually delineated by pathologists to ensure reliable tumor region analysis. Consistent segmentation and fluorescence thresholds were applied to maintain the reliability of the analysis across samples. tsMHC-I/II expression intensity was standardized to the average fluorescence intensity (MFI) in the tumor epithelial region, using twice the intensity of the negative control as the threshold. Meanwhile, the tsMHC-I/II ratio was calculated as the proportion of tsMHC-I/II-positive cells within the total tumor cells of each patient and the density of CD4 and CD8 was calculated as the ratio of positive cells to total cells. Spatial distance analysis involved calculating the Euclidean distance (µm) to the nearest cells of the corresponding subgroup from the tumor epithelial region.

### Flow cytometry

For each sample, 1 million tumor cells were collected, washed, and incubated with an appropriate concentration of FITC anti-human HLA-A, B, C antibody (clone: W6/32, Biolegend, 311404) and PE anti-human HLA-DR, DP, DQ antibody (clone: Tü39, Biolegend, 361716) at room temperature for 30 min. After incubation, the cells were washed once and analyzed using a CytoFLEX flow cytometer (Beckman).

### SiRNA transfection

The transfection procedure for the cell lines in this study was adapted from previous research [53]. Briefly, 5 µl of Lipofectamine 3000 Transfection Reagent (ThermoFisher) and 5 µl of si-KAT2B were added to 500 µl of Opti-MEM reduced serum medium (ThermoFisher). The mixture was gently mixed and incubated at room temperature for 15 min, then slowly added dropwise to cell culture medium without FBS. The sequence of KAT2B was listed in Table S1.

### PDO establishment and culture

In this study, the culture medium formulation and protocol for PDOs were adapted from Johanna et al.‘s research [54]. Briefly, fresh TNBC tumor samples were washed with physiological saline, minced into small pieces, and dissociated into a single-cell suspension using a tumor dissociation kit (Miltenyi, 130-095-929) according to the manufacturer’s instructions. Red blood cells were removed by treating the suspension with red blood cell lysis buffer (Solarbio) for 2 min, followed by filtration through a 100 μm cell strainer (Corning). Based on cell count, Basement Membrane Extract (BME) (R&D, 3533-010-02) was added, and the cells were seeded into low-attachment culture plates (Corning). For PDO passaging, the BME was first digested using a harvesting solution (R&D, 3700-100-01), followed by dissociation of the PDOs with TrypLE Express Enzyme (Invitrogen). The dissociated cells were then mixed with BME at a ratio of 1:2 to 1:3 and reseeded into new culture plates.

### PDO co-culture in vitro

Isolation and Culture of Peripheral Blood CD44 + CD4 + Effector T Cells: Peripheral blood from PDO-paired patients was subjected to gradient centrifugation using Ficoll-Paque (Cytiva, 17144002) according to the protocol to obtain peripheral blood mononuclear cells (PBMCs). Red blood cells were removed by treating the suspension with red blood cell lysis buffer (Solarbio) for 2 min. CD4 + T cells were isolated using a CD4 + T Cell Isolation Kit (Miltenyi, 130-096-533) by incubating with antibodies and magnetic beads as per the protocol, followed by separation through an LS column. The isolated CD4 + T cells were cultured in vitro for 5 days in T cell expansion medium (Stemcell) supplemented with 100 U/ml IL-2 (PeproTech) and Human CD3/CD28 T Cell Activator (Stemcell). Subsequently, CD4 + T cells were incubated with anti-human FITC CD4 + antibody, and CD44 + CD4 + effector T cells were sorted using Fluorescence-Activated Cell Sorting (FACS).

PDO Immune Cell Co-culture: PDOs were harvested by removing BME with a harvesting solution and combined with CD44 + CD4 + effector T cells at a 1:10 ratio to establish the co-culture system. The culture medium was prepared by mixing PDO medium and T cell medium at a 1:1 ratio. Prior to co-culture, PDOs were labeled with CellTrace Violet fluorescence dye (Invitrogen, C34557), while CD4 + T cells were labeled with CellTrace Far Red fluorescence dye (Invitrogen, C34564).

### ATAC-seq analysis

The ATAC-seq data for the TNBC cell lines were sourced from Harris et al.‘s study [47] (GSE223182). The raw sequencing data underwent quality assessment using FastQC (V0.12.0). Subsequently, data trimming was performed using Skewer [55], and the distribution of reads relative to transcription start sites (TSS), gene bodies, and peak summits was calculated using the deepTools software [56]. The reads data were visualized using the Integrative Genomics Viewer (IGV).

### Estimation of immune cell subtypes in tumor

We evaluated the proportions of immune cell subgroups in the FUSCC RNA-seq cohort, TCGA, and I-SPY2 tumor samples using the CIBERSORTx tool (https://cibersortx.stanford.edu).

### Statistical analysis

The DFS and OS analyses were performed by Cox Kaplan-Meier method. The p-values within the same analysis group were adjusted by Benjamini-Hochberg method. Multivariate Cox regression models were used to adjust for potential confounding factors by including variables with a univariate regression p-value < 0.05. For subgroup analyses, all relevant clinical factors were incorporated as covariates in the multivariate Cox regression analysis. Multivariate logistic regression analysis was similarly conducted by including variables with univariate logistic regression p-values < 0.05.

A one-sided t-test was used to compare the T cell subgroups between the two groups in the tumor. The proportions of pCR and RD in the NeoTRIP cohort were compared by a one-sided chi-square test. Data quality was checked prior to analysis. For continuous variables, distributions were examined, and appropriate statistical methods robust to or accounting for potential outliers were employed where necessary. Outliers were investigated for potential errors, but data points were not removed unless confirmed to be erroneous. Since this study is retrospective, a power analysis is not required. All analyses were performed by R (version 4.2.1). Intergroup t-tests were conducted by GraphPad Prism (version 9.4.1), and results were presented as Mean values with 95% CI. Statistical significance was defined as ns (no significant), *P ≤ 0.05, **P ≤ 0.01, ***P ≤ 0.001.

## Electronic supplementary material

Below is the link to the electronic supplementary material.

Supplementary Material 1

Table 1 Clinical characteristics of FUSCC exploration cohort

Table 2 Clinical characteristics of FUSCC TNBC validation cohort

## Data Availability

No datasets were generated or analysed during the current study.
