# Supplementary material for: Tumor-specific MHC-II guides anthracycline exemption and immunotherapy benefit in breast cancer
Source: Biomark Res. 2025 Jun 10;13:83. doi: 10.1186/s40364-025-00797-9 (PMC12150567; doi:10.1186/s40364-025-00797-9)
Supplement: Supplementary file 1 — Supplementary Material 1 [file 40364_2025_797_MOESM1_ESM.zip › Supplementary Material-Revised/Supplemental Method.docx]

**Supplemental Method**

**Cell Culture**

In this study, the MDA-MB-231, MDA-MB-157, MDA-MB-468, and BT-549 cell lines were all purchased from the American Type Culture Collection and cultured in an environment with 5% CO2 at 37°C.

**RNA Analysis**

Real-time quantitative PCR was conducted by TB Green Fast qPCR Mix (TAKARA) in 7900HT PCR System (Applied Biosystems). And the primer sequences are listed in Table S1.

**GSEA and ssGSEA Analysis**

GSEA was conducted by GSEA software (V 4.4.0). Pathways were selected using GO.BP.v2024.1.Hs.symbols.gmt and GO.MF.v2024.1.Hs.symbols.gmt from the MSigDB database (https://www.gsea-msigdb.org/gsea/msigdb/index.jsp). Analysis conditions were set with gene sets ranging from 10 to 300 and 1000 permutations. The GSEA results were visualized for enrichment using aPEAR in R (V4.0.3)^[1]^. The ssGSEA analysis was performed using the GSVA package (2.0.6) with default parameters This implementation ensures robust and reproducible enrichment scoring across samples including FUSCC RNA-seq cohort, TCGA, and I-SPY2 tumor samples. In the databases above, patients’ pathway expressions were grouped into high-expression and low-expression categories based on the median value. The ssGSEA pathway signatures analyzed in this study are detailed in Table S2.

**ChIP**

In this study, the Pierce Magnetic ChIP Kit (#26157, ThermoFisher) was utilized, and the ChIP-qPCR procedure was performed according to the manufacturer's protocol. Briefly, 2×10^8 viable cells were crosslinked with 1% formaldehyde for 10 minutes, followed by quenching with glycine. After washing, cells were resuspended in lysis buffer. An aliquot of the lysate was separated for the Input group. Chromatin was fragmented by sonication, and gel electrophoresis confirmed DNA fragment sizes within the 200-1000bp range. Appropriate amounts of ChIP antibodies or IgG negative control antibodies were added and incubated overnight at 4°C. The following day, pre-equilibrated Protein A/G magnetic beads were added and incubated at room temperature for 1-3 hours. Beads were subsequently washed with washing buffer, and protein-DNA complexes were eluted using elution buffer with two 30-minute incubations at 65°C. All eluates were collected. Proteinase K was added and incubated overnight at 65°C to remove proteins. Finally, antibody-enriched DNA was purified using DNA purification columns.

The ChIP antibodies used in this study included: KAT2B: PCAF Rabbit mAb (Clone C14G9, 3378, 1:25, CST); H3K14ac: Acetyl-Histone H3 (Lys14) Rabbit mAb (Clone D4B9, 7627, 1:50, CST); H4K8ac: Rabbit Recombinant Monoclonal Histone H4 acetyl K8 antibody (Clone EP1002Y, ab45166, 2μg for 25μg of chromatin, Abcam); IgG: Rabbit Recombinant Monoclonal IgG antibody (Clone EPR25A, ab172730, 2μg for 25μg of chromatin, Abcam). Primers used for promoter truncation in this study are detailed in Table S1.

Western blot

The Western blot methodology in this study followed protocols from previous research^[2]^. Briefly, cells were thoroughly lysed with RIPA lysis buffer containing protease inhibitors, followed by centrifugation at 12,000 rpm for 15 minutes at 4°C. Protein concentration in the supernatant was quantified using the BCA method. Samples were then mixed with SDS loading buffer and denatured at 95°C. Protein samples were separated by electrophoresis on 7.5% or 10% SDS-polyacrylamide gels. Proteins were transferred from the gel to PVDF membranes using wet transfer in methanol buffer. Membranes were subsequently blocked with TBST buffer containing 5% skim milk powder at room temperature for 1 hour, followed by incubation with the corresponding primary antibodies overnight at 4°C. The following day, after washing, horseradish peroxidase-conjugated secondary antibodies were added and incubated at room temperature for 1 hour. After washing, HRP signals were detected using an enhanced chemiluminescence (ECL) kit.

The antibodies used in this study were as follows: KAT2B: PCAF Rabbit mAb (Clone C14G9, 3378, 1:25, CST); CIITA: Rabbit Recombinant Monoclonal CIITA Antibody (ab28349, 1:500, Abcam); HLA-DR: Rabbit Recombinant Monoclonal HLA-DR Antibody (Clone EPR3692, ab92511, 1:1000, Abcam); Beta-actin: Beta Actin Monoclonal Mouse antibody (Clone 2D4H5, 66009-1-Ig, 1:20000, Proteintech).

**Reference**

[1] Kerseviciute I, Gordevicius J. *aPEAR* : an R package for autonomous visualization of pathway enrichment networks[J]. KELSO J. Bioinformatics, 2023, 39(11): btad672.

[2] Wang Z, Yang L, Wu P, Li X, Tang Y, Ou X, Zhang Y, Xiao X, Wang J, Tang H. The circROBO1/KLF5/FUS feedback loop regulates the liver metastasis of breast cancer by inhibiting the selective autophagy of afadin[J]. Molecular Cancer, 2022, 21(1): 29.
