# Supplementary figures and images for: Tumor-specific MHC-II guides anthracycline exemption and immunotherapy benefit in breast cancer
Source: Biomark Res. 2025 Jun 10;13:83. doi: 10.1186/s40364-025-00797-9 (PMC12150567; doi:10.1186/s40364-025-00797-9)

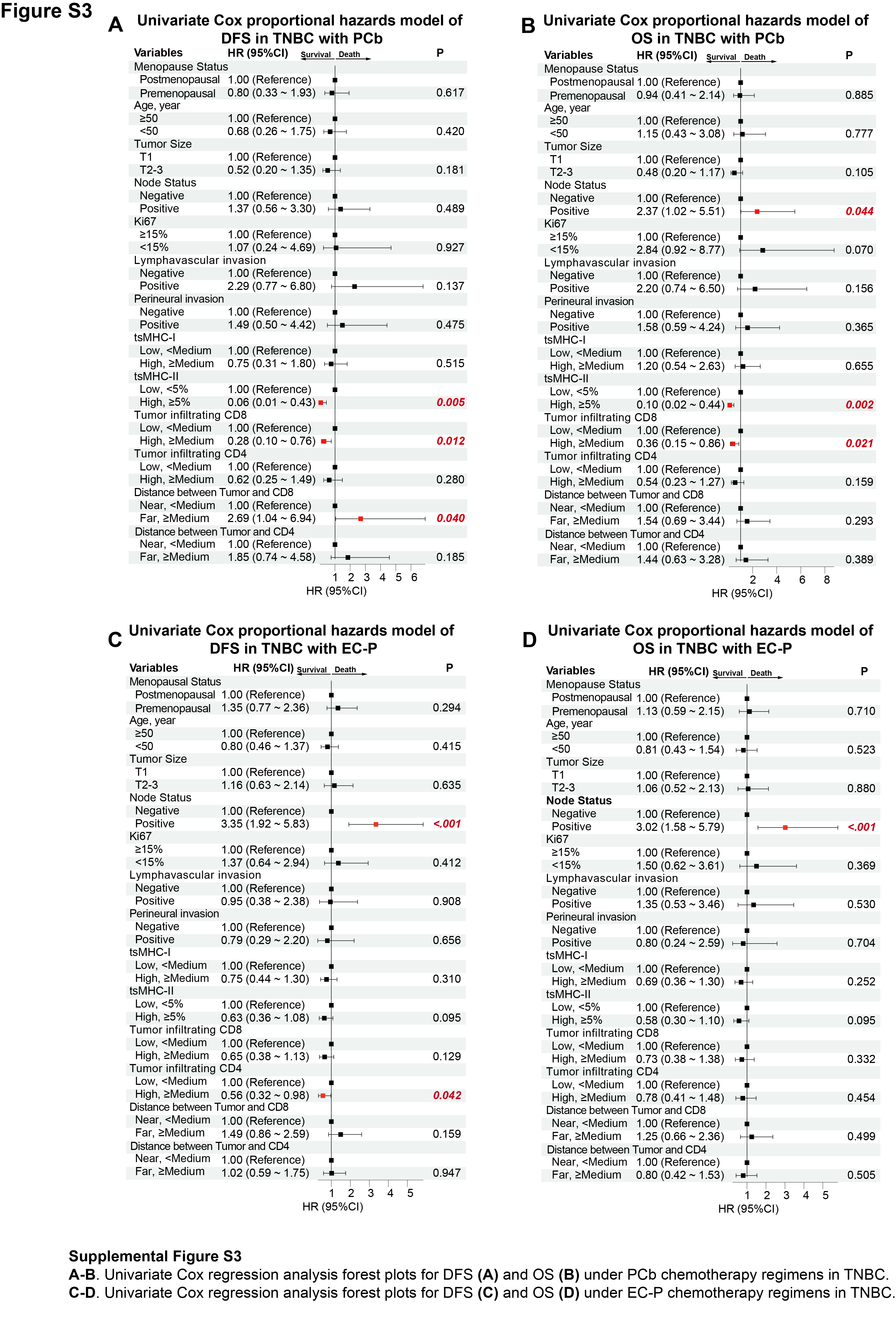

Supplement: Supplementary file 1 — Supplementary Material 1 [file 40364_2025_797_MOESM1_ESM.zip › Supplementary Material-Revised/Figure S3.png]

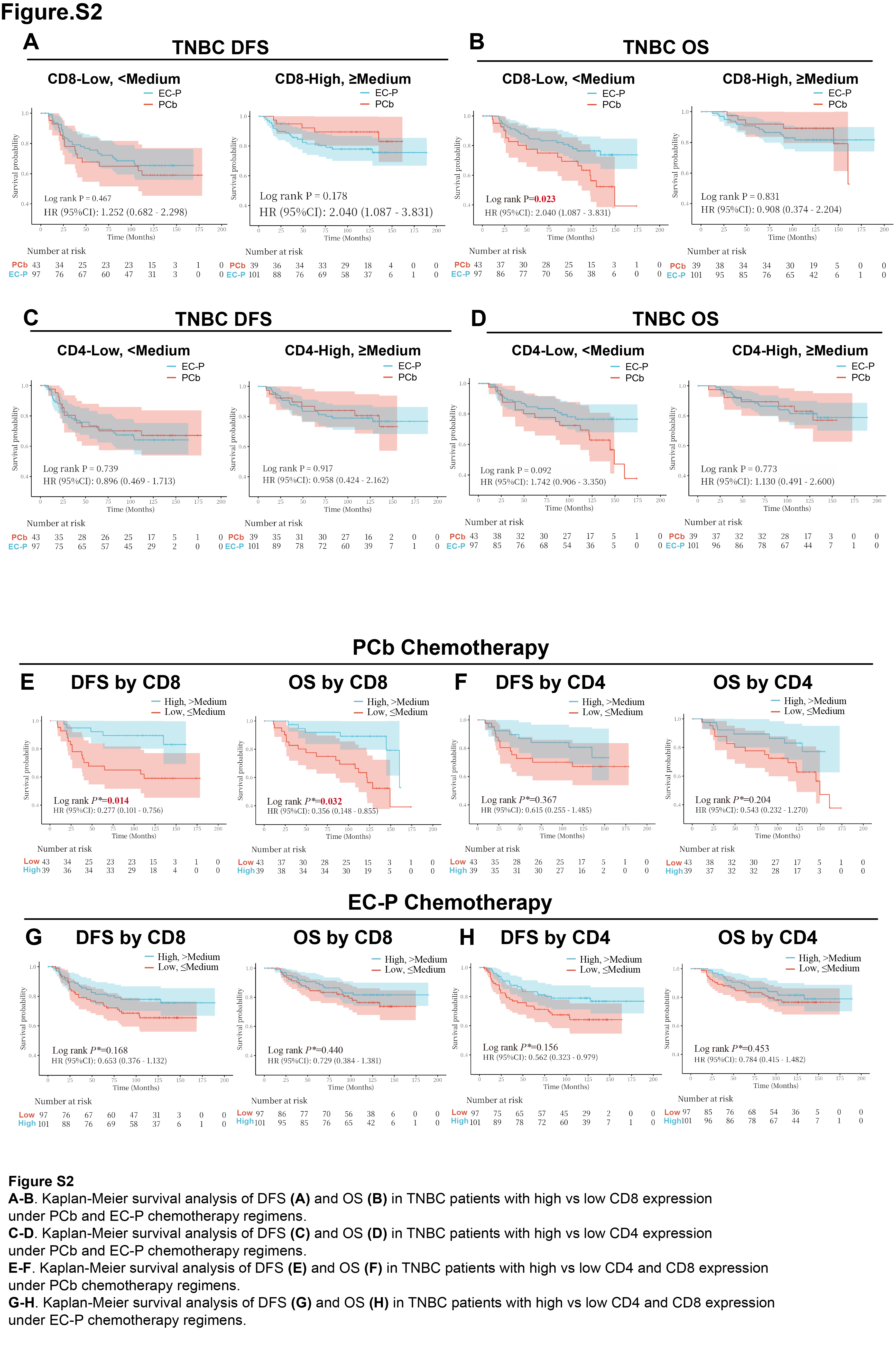

Supplement: Supplementary file 1 — Supplementary Material 1 [file 40364_2025_797_MOESM1_ESM.zip › Supplementary Material-Revised/Figure S2.png]

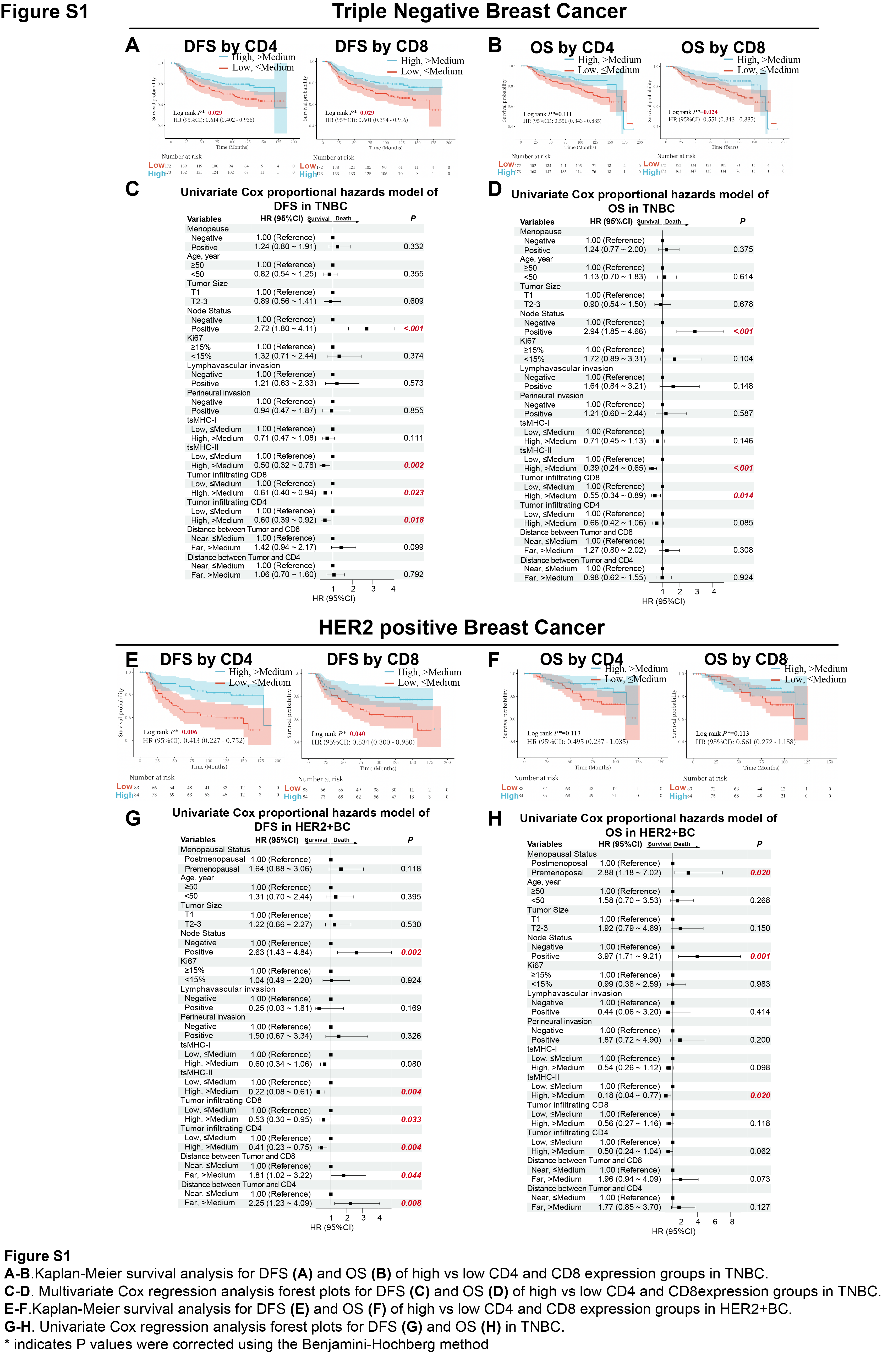

Supplement: Supplementary file 1 — Supplementary Material 1 [file 40364_2025_797_MOESM1_ESM.zip › Supplementary Material-Revised/Figure S1.png]

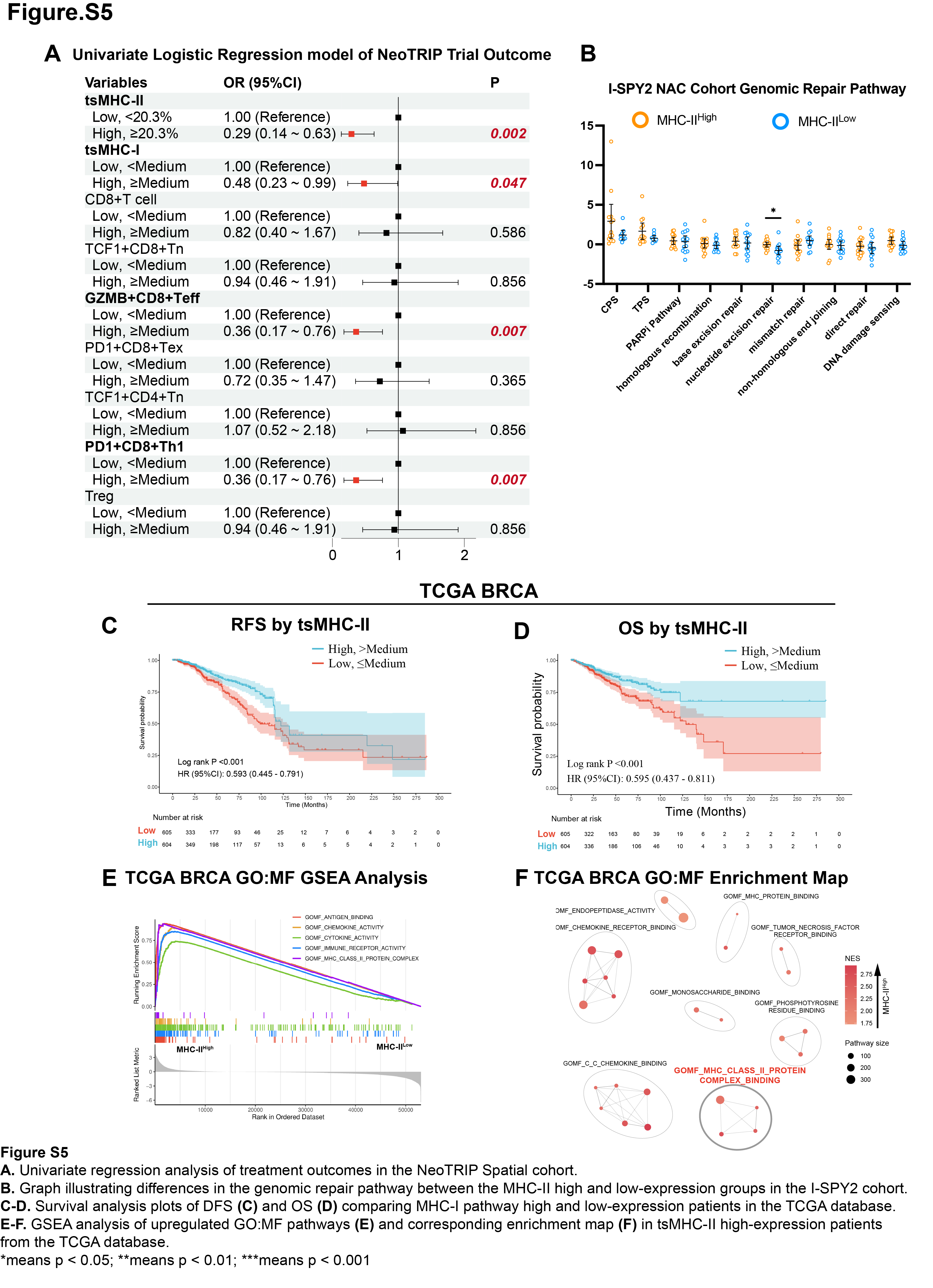

Supplement: Supplementary file 1 — Supplementary Material 1 [file 40364_2025_797_MOESM1_ESM.zip › Supplementary Material-Revised/Figure S5.png]

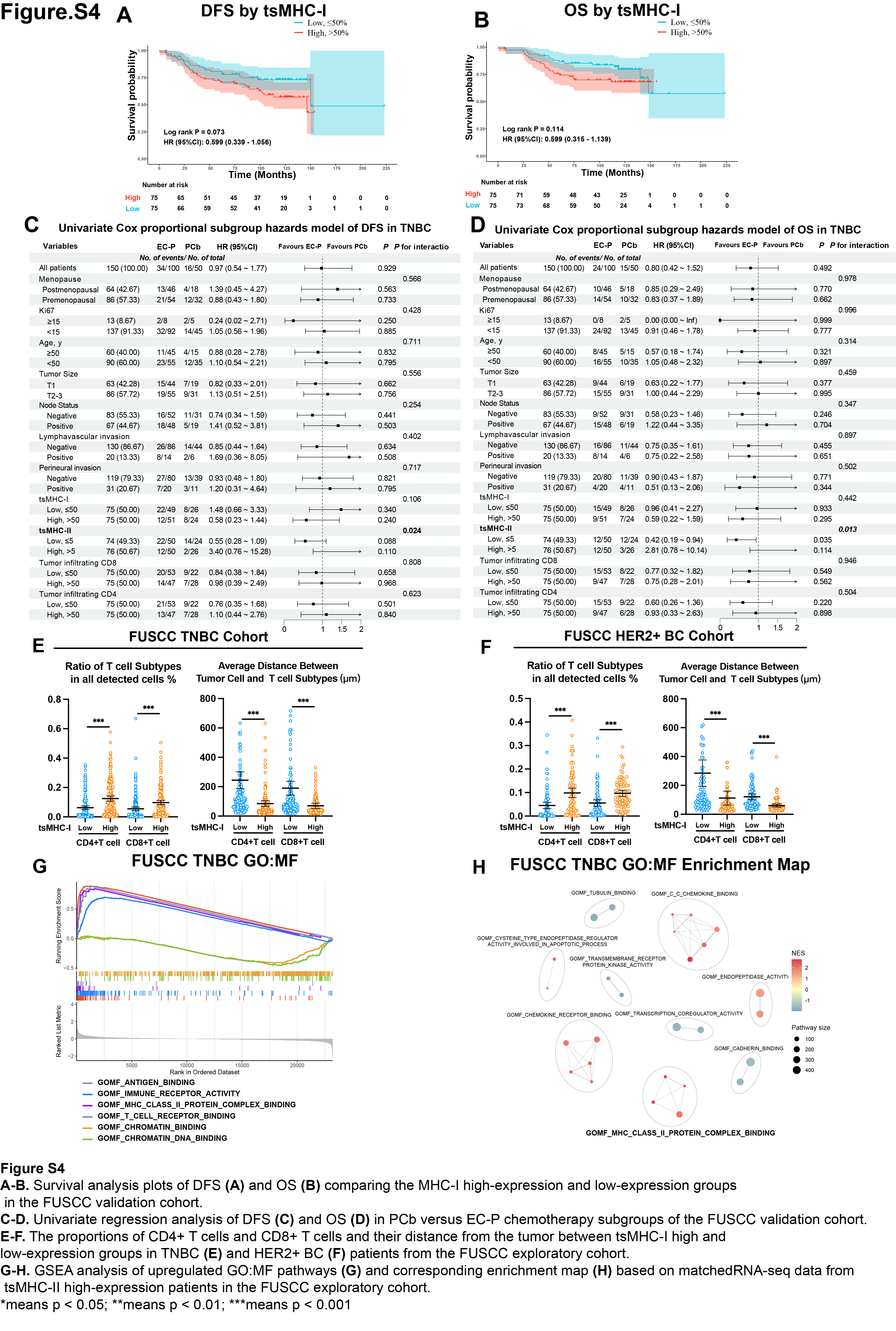

Supplement: Supplementary file 1 — Supplementary Material 1 [file 40364_2025_797_MOESM1_ESM.zip › Supplementary Material-Revised/Figure S4.png]

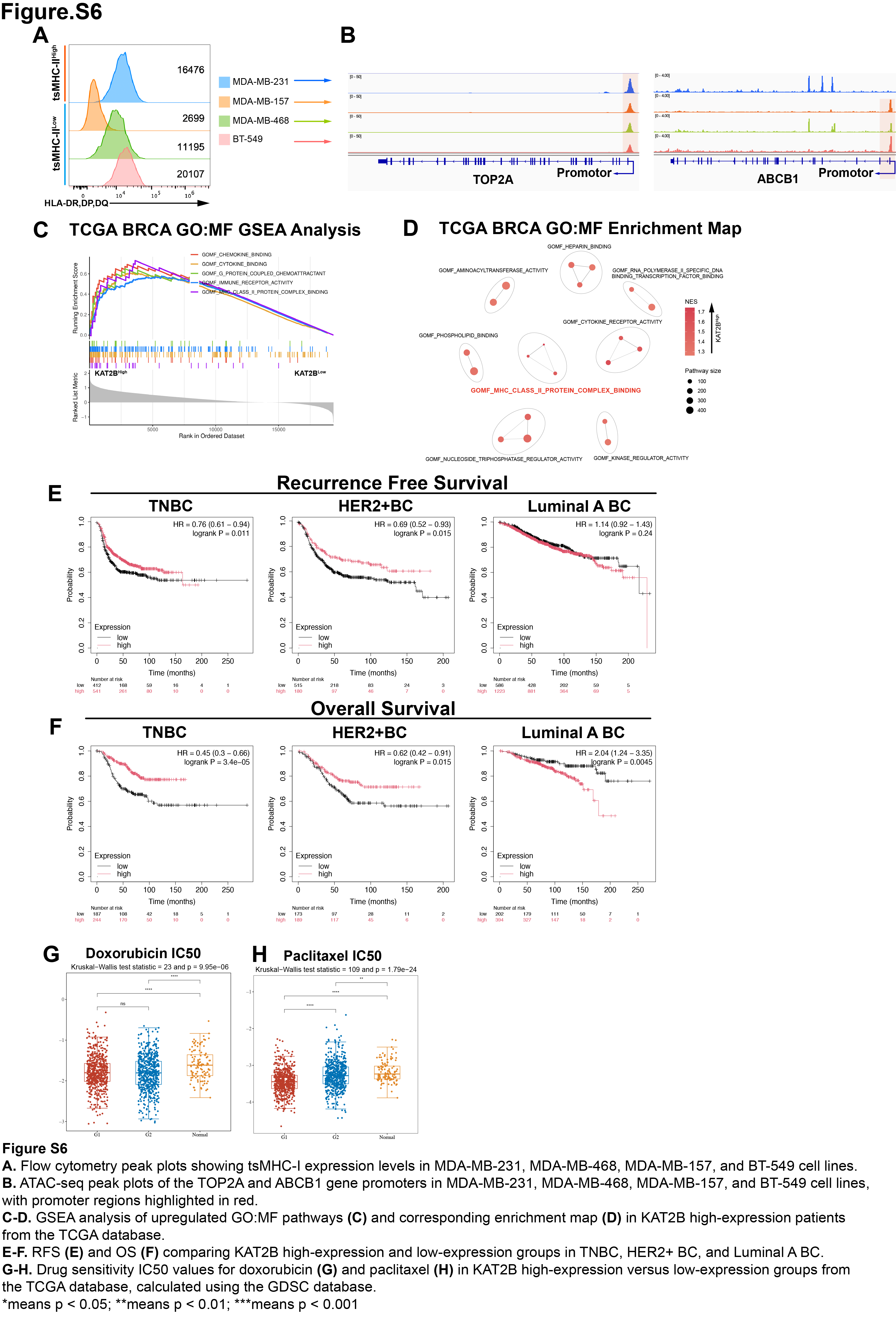

Supplement: Supplementary file 1 — Supplementary Material 1 [file 40364_2025_797_MOESM1_ESM.zip › Supplementary Material-Revised/Figure S6.png]
