# Supplementary material for: Tumor-specific MHC-II guides anthracycline exemption and immunotherapy benefit in breast cancer
Source: Biomark Res. 2025 Jun 10;13:83. doi: 10.1186/s40364-025-00797-9 (PMC12150567; doi:10.1186/s40364-025-00797-9)
Supplement: Supplementary file 2 — Table 1 Clinical characteristics of FUSCC exploration cohort [file 40364_2025_797_MOESM2_ESM.pdf]

Table 1. Clinical characteristics of FUSCC Exploration Cohort.

Characteristic	HER2+BC (n=167), No. (%)	TNBC (n=345), No. (%)
Menopause		
Postmenopausal Status	63 (38)	140 (41)
Premenopausal Status	104 (62)	205 (59)
Age, y		
<50	55 (33)	129 (37)
≥50	112 (67)	216 (63)
Tumor size		
0-20mm	52 (31)	87 (25)
20-50	108 (65)	247 (72)
>50	7 (4)	11 (3)
Nodal status		
Negative	84 (50)	233 (68)
1-3 positive	34 (20)	62 (18)
4-9 positive	25 (15)	32 (9)
>9 positive	24 (14)	18 (5)
Histologic type		
Ductal carcinoma in situ	0 (0)	0(0)
Infiltrating ductal carcinoma	167 (100)	345 (100)
Vascular tumor thrombus		
Negative	155 (93)	310 (90)
Positive	12 (7)	35 (10)
Perineural invasion		
Negative	149 (89)	310 (90)
Positive	18 (11)	35 (10)
ER		
Negative (0-10%)	162 (97)	345 (100)
Positive (>10%)	5 (3)	0 (0)
PR		
Negative (0-10%)	123 (74)	345 (100)

Characteristic	HER2+BC (n=167), No. (%)	TNBC (n=345), No. (%)
Positive (>10%)	44 (26)	0 (0)
HER2		
Negative (IHC 0, 1+ and/or FISH <2.0)	0 (0)	345 (100)
Positive (IHC 3+ or FISH ≥2.0)	167 (100)	0 (0)
Ki67		
Low (<15%)	33 (20)	36 (10)
High (≥15%)	90 (54)	260 (75)
Unknown	44 (26)	49 (14)
Chemo		
EC	26 (16)	52 (15)
PCb	34 (20)	82 (22)
EC-P	87 (52)	198 (57)
Other	20 (12)	13 (4)
Radiation therapy		
No	108 (65)	224 (65)
Yes	59 (35)	121 (35)
All DFS events	50 (30)	90 (26)
Breast cancer recurrence	11 (7)	34 (10)
Distant recurrence	29 (17)	44 (13)
Second non–breast primary cancer	10 (6)	12 (3)
All deaths	32 (20)	74 (21)
Breast cancer-related	29 (17)	59 (17)
Second non-breast cancer	2 (1)	4 (1)
Not cancer-related	1 (<1)	8 (2)
Unknown	0 (0)	3 (<1)
