# Supplementary material for: Tumor-specific MHC-II guides anthracycline exemption and immunotherapy benefit in breast cancer
Source: Biomark Res. 2025 Jun 10;13:83. doi: 10.1186/s40364-025-00797-9 (PMC12150567; doi:10.1186/s40364-025-00797-9)
Supplement: Supplementary file 3 — Table 2 Clinical characteristics of FUSCC TNBC validation cohort [file 40364_2025_797_MOESM3_ESM.pdf]

Table 2. Clinical characteristics of FUSCC TNBC Validation Cohort

Characteristic	EC-P (n=100), No. (%)	PCb (n=50), No. (%)
Menopause		
Postmenopausal Status	46 (46)	19 (38)
Premenopausal Status	54 (54)	31 (62)
Age, y		
<50	55 (55)	15 (30)
≥50	45 (45)	35 (70)
Tumor size		
0-20mm	44 (44)	10 (20)
20-50	55 (55)	30 (60)
>50	1 (1)	10 (20)
Nodal status		
Negative	52 (52)	31 (62)
1-3 positive	31 (31)	10 (20)
4-9 positive	10 (10)	4 (8)
>9 positive	7 (7)	0 (0)
Histologic type		
Ductal carcinoma in situ	0 (0)	0(0)
Infiltrating ductal carcinoma	100 (100)	50 (100)
Vascular tumor thrombus		
Negative	86 (86)	44 (88)
Positive	14 (14)	6 (12)
Perineural invasion		
Negative	80 (80)	39 (78)
Positive	20 (20)	11 (22)
ER		
Negative (0-10%)	100 (100)	50 (100)
Positive (>10%)	0 (0)	0 (0)
PR		
Negative (0-10%)	100 (100)	50 (100)

Characteristic	EC-P (n=100), No. (%)	PCb (n=50), No. (%)
Positive (>10%)	0 (0)	0 (0)
HER2		
Negative (IHC 0, 1+ and/or FISH <2.0)	100 (100)	50 (100)
Positive (IHC 3+ or FISH ≥2.0)	0 (0)	0 (0)
Ki67		
Low (<15%)	7 (7)	5 (10)
High (≥15%)	93 (93)	45 (90)
Chemo		
EC	0 (0)	0 (0)
PCb	0 (0)	50 (100)
EC-P	100 (100)	0 (0)
Other	0 (0)	0 (0)
Radiation therapy		
No	55 (55)	36 (72)
Yes	45 (45)	14 (28)
All DFS events	34 (34)	17 (34)
Breast cancer recurrence	6 (6)	2 (4)
Distant recurrence	26 (26)	12 (24)
Second non–breast primary cancer	2 (2)	3 (6)
All deaths	32 (32)	15 (30)
Breast cancer-related	22 (22)	13 (26)
Second non-breast cancer	1 (1)	1 (2)
Not cancer-related	1 (1)	1 (2)
Unknown	1 (1)	0 (0)
